# Mathematical modelling of the dynamics of image-informed tumor habitats in a murine model of glioma

**DOI:** 10.1038/s41598-023-30010-6

**Published:** 2023-02-20

**Authors:** Kalina P. Slavkova, Sahil H. Patel, Zachary Cacini, Anum S. Kazerouni, Andrea L. Gardner, Thomas E. Yankeelov, David A. Hormuth

**Affiliations:** 1 Department of Physics, The University of Texas at Austin, Austin, TX USA; 2 Department of Computer Science, Case Western Reserve University, Cleveland, OH USA; 3 Department of Bioengineering, University of Illinois, Urbana-Champaign, IL USA; 4 Department of Radiology, The University of Washington, Seattle, WA USA; 5 Department of Biomedical Engineering, The University of Texas at Austin, Austin, USA; 6 Department of Diagnostic Medicine, The University of Texas at Austin, Austin, TX USA; 7 Department of Oncology, The University of Texas at Austin, Austin, TX USA; 8 The Oden Institute for Computational Engineering and Sciences, The University of Texas at Austin, 201 E 24th Street, Austin, TX 78712 USA; 9 Livestrong Cancer Institutes, The University of Texas at Austin, Austin, TX USA; 10 Department of Imaging Physics, The University of Texas MD Anderson Cancer Center, Houston, TX USA

**Keywords:** Cancer imaging, Cancer models, Tumour heterogeneity, Computational science

## Abstract

Tumors exhibit high molecular, phenotypic, and physiological heterogeneity. In this effort, we employ quantitative magnetic resonance imaging (MRI) data to capture this heterogeneity through imaging-based subregions or “habitats” in a murine model of glioma. We then demonstrate the ability to model and predict the growth of the habitats using coupled ordinary differential equations (ODEs) in the presence and absence of radiotherapy. Female Wistar rats (N = 21) were inoculated intracranially with 10^6^ C6 glioma cells, a subset of which received 20 Gy (N = 5) or 40 Gy (N = 8) of radiation. All rats underwent diffusion-weighted and dynamic contrast-enhanced MRI at up to seven time points. All MRI data at each visit were subsequently clustered using *k*-means to identify physiological tumor habitats. A family of four models consisting of three coupled ODEs were developed and calibrated to the habitat time series of control and treated rats and evaluated for predictive capability. The Akaike Information Criterion was used for model selection, and the normalized sum-of-square-error (SSE) was used to evaluate goodness-of-fit in model calibration and prediction. Three tumor habitats with significantly different imaging data characteristics (*p* < 0.05) were identified: high-vascularity high-cellularity, low-vascularity high-cellularity, and low-vascularity low-cellularity. Model selection resulted in a five-parameter model whose predictions of habitat dynamics yielded SSEs that were similar to the SSEs from the calibrated model. It is thus feasible to mathematically describe habitat dynamics in a preclinical model of glioma using biology-based ODEs, showing promise for forecasting heterogeneous tumor behavior.

## Introduction

Gliomas are generally a neoplastic growth of the glial cells, which are cells that serve supportive functions in the central and peripheral nervous systems^1^. High-grade gliomas, such as glioblastoma, are extremely aggressive forms that have a high recurrence rate—despite treatment combinations of surgery, radiotherapy, and chemotherapy—and a 5-year-survival rate of only 7.1%^2^. It is well established that the aggressiveness of glioblastomas is largely attributed to intra-tumor heterogeneity^3^; namely, phenotypic and genotypic differences that lead to variations in proliferation rates and treatment sensitivities across the tumor^3,4^. Intra-tumor heterogeneity arises from a diverse array of tumor sub-populations as well as stromal cells that infiltrate the tumor and become a part of the tumor microenvironment^4^. As tumor cells divide and evolve, tumors become more heterogeneous, leading to a differential response to therapies and, ultimately, treatment resistance^5^. In addition to heterogeneity arising from genotypic and phenotypic variables, heterogeneity in tumor vasculature also contributes to the mixed tumor landscape and substantially informs treatment outcomes. Previous work has described tumor heterogeneity through the investigation of tumor subregions, termed habitats^6^, with unique physiological features stemming from properties of the tumor vasculature and cellularity^7,8^. These habitats can be non-invasively identified through multiparametric imaging for three-dimensional (3D) analysis of tumor heterogeneity.

Common methods for evaluating tumor biology involve invasive procedures, such as biopsies or tumor excisions^9^. These techniques are susceptible to sampling error given that they interrogate only a small portion of a heterogeneous tumor, making it unlikely that they provide an accurate description of the whole lesion^9,10^. Additionally, invasive methods are fundamentally limited in their ability to characterize dynamic events such as vascular perfusion or metabolic activity. Conversely, quantitative magnetic resonance imaging (MRI) allows for noninvasive 3D measurements of physiological tissue characteristics and, therefore, enables measurement at multiple time points^11^. In particular, diffusion-weighted MRI (DW-MRI)^12^ and dynamic contrast-enhanced (DCE-) MRI^13^ are two prominent acquisition techniques that quantitatively assess tissue cellularity and vascularity, respectively, which are tumor features that are known to be dramatically altered in high-grade glioma^14^.

Tissue cellularity is quantified via DW-MRI, which is sensitive to the movement of water molecules within tissue. DW-MRI can be analyzed to yield an estimate of the apparent diffusion coefficient (*ADC*, units of mm^2^/s) of water in tissue, which has been established as having an inverse relationship with the level of cellularity in tumors^12^. Increased cellularity results in an increase in the number of barriers to water movement and therefore a reduction in the *ADC*. Decreased *ADC* values have been associated with poorer survival^15^, while increased *ADC* values have been associated with positive response to chemotherapy^15–17^.

In DCE-MRI, a subject is injected with a gadolinium-based contrast agent, and images are serially collected before and after contrast delivery. This dynamic data can then be analyzed with (for example) the Kety–Tofts perfusion model^18^ to yield two perfusion parameters: the volume transfer constant, *K*^*trans*^ (units of min^−1^, a mixed measured of vessel permeability and perfusion), and the extravascular/extracellular volume fraction, *v*_*e*_ (a unitless measure of the fraction of the volume that is external to cells and vessels)^13^. *K*^*trans*^ and *v*_*e*_ have been found to characterize vascular properties of tumors^14^, and repeatability of these perfusion parameters has been assessed in healthy subjects and patients with high-grade glioma^15,19^. Additionally, Aydin et al.^14^ found that *K*^*trans*^ and *v*_*e*_ showcase a strong positive correlation with brain tumor grade.

Typical approaches for employing MRI to quantify intra-tumoral heterogeneity include histogram analysis of quantitative parameter maps resulting from DW- and DCE-MRI data^20,21^. This approach, however, eliminates the spatial information available from the imaging data. Other efforts have been made to apply texture analysis and machine learning techniques to identify and enhance heterogeneity without loss of spatial information^22,23^, but these methods assume that tumors are well-mixed heterogeneous bodies rather than possessing distinct sub-regions^6^, especially in cases of high-grade gliomas with surrounding edema^24^. Recently, Syed et al. demonstrated the ability to identify three distinct tumor habitats using multiparametric preclinical MRI data that were subsequently histologically validated in murine models of breast cancer.^7^ A follow-up study by the same team showed that two distinct tumor phenotypes emerged in which one phenotype exhibited greater sensitivity to treatment^25^.

Because of the high rate of recurrence of high-grade glioma, treatment strategies are often tailored to each independent case^25^. In recent years, we^26–28^ and others^25,29,30^ have leveraged imaging data to personalize partial differential equation (PDE) models, such as reaction–diffusion models, of glioma progression that predict glioma response to radiotherapy and provide an avenue for non-invasive treatment optimization with the goal of improving patient survival. These PDE models have several benefits over simpler ordinary differential equation (ODE) models, such as providing spatially resolved predictions. It is non-trivial, however, to build parsimonious multi-species PDE models with limited assumptions on how the tumor habitats interact. Moreover, image-informed PDE models require additional image preprocessing steps, such as registration of longitudinal imaging data. ODE models are conducive to fast data-driven modeling of tumor growth for treatment optimization where assumptions can be relaxed and data alone can be relied upon for parameterizing the modeling framework. While ODE models have been employed by others^25,31,32^, these models either do not incorporate non-invasive imaging data or do not account for intra-tumor heterogeneity.

Characterization and analysis of tumor heterogeneity via quantitative imaging and mathematical modeling could enable treatment optimization to maximize the effect of therapy across the entire tumor. In this work, we developed a data-driven ODE model to describe the dynamics of tumor habitats identified via quantitative MRI with and without radiotherapy in a murine model of glioma. We demonstrate that the identified habitats corroborate previous results in murine models of cancer, in which each habitat exhibits a unique combination of vascularity and cellularity. A family of mathematical models consisting of ODEs is calibrated to the dynamic habitat data and evaluated using a normalized sum-of-square-error as an error metric. After quantitatively selecting the most parsimonious model, we investigated the ability of the chosen model to predict the growth of the tumor habitats across time using two separate approaches. We show that the selected model makes successful predictions of the habitat dynamics that agree with the calibrated model outputs.

## Methods

### Animal model and magnetic resonance imaging

All experimental details were previously reported in Hormuth et al.^26,27^ For brevity, we include salient details of the methods below (see Supplemental Methods for complete details). All experimental procedures were approved by our Institutional Animal Care and Use Committee and were performed in accordance with relevant guidelines and regulations. This study is reported in accordance with ARRIVE guidelines (https://arriveguidelines.org/). Female Wistar rats (N = 21) were inoculated intracranially with C6 glioma cells (1 × 10^5^) through stereotaxic injection. The C6 glioma line was chosen as it is widely used in preclinical studies in neuro-oncology and in mathematical modelling due to its predictable and reliable growth patterns^33^. Of the 21 rats, 8 received no treatment and were assigned to the control group, 8 received 40 Gy of radiation, and 5 received 20 Gy of radiation. Rats were imaged with DCE-MRI and DW-MRI protocols beginning 10 days post-inoculation and then every 1–2 days after that for a total of 5–7 MRI data sets per animal.

### Multiparametric MRI analysis

**Figure 1:**
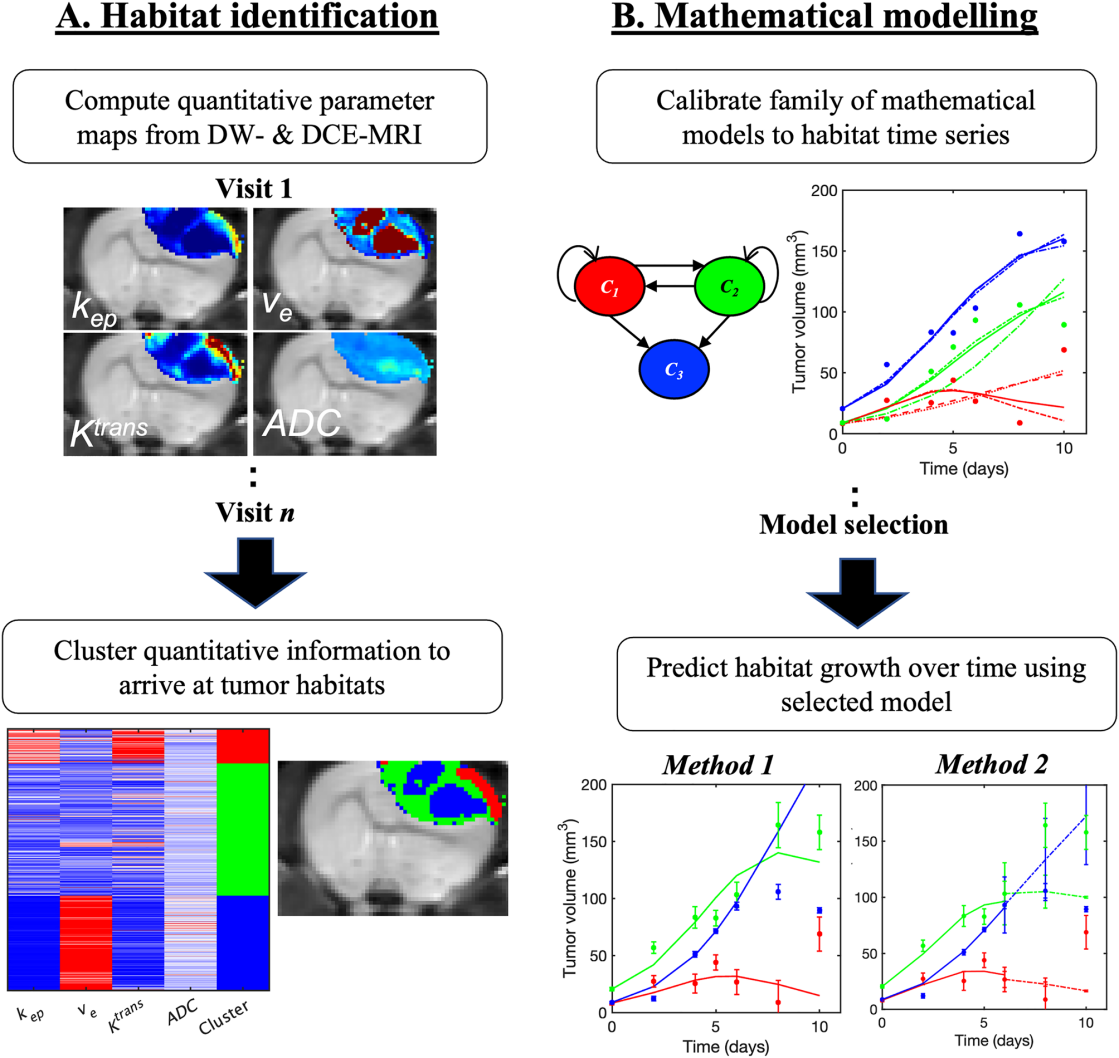
Habitat identification and modelling pipeline. (**A**) DCE-MRI and DW-MRI data were acquired for each animal over multiple days. The multiparametric MRI data were analyzed to generate four parameter maps (i.e., *k*_*ep*_, *v*_*e*_, *K*^*trans*^, ADC) across the tumor ROI (top left). The quantitative information at each voxel at all imaging visits for all animals was pooled together into one conglomerate feature matrix and then clustered to identify three tumor habitats with unique physiologies: high-vascularity high-cellularity, low-vascularity high-cellularity, and low-vascularity low-cellularity (bottom left). (**B**) Once the habitats were identified, each rat’s tumor was divided into three habitat time series that could be analyzed by a family of mathematical models based on three compartments and the transition rates between them (top right). We then tested the ability of the most parsimonious model to predict habitat dynamics through leave-one-out (Method 1) and bootstrapping (Method 2) analyses (bottom right).

The DW- and DCE-MRI data from all control and treated rats were analyzed to extract four quantitative MRI parameters that would be clustered to identify tumor habitats (Fig. 1A). For each rat, the tumor regions of interest (ROIs) were manually segmented using the DCE-MRI data. Nonlinear least squares methods were then used to fit the standard Kety–Tofts perfusion model^13,18,34^ to the DCE-MRI data within the tumor ROI, yielding three pharmacokinetic parameters: volume transfer constant (*K*^*trans*^, in units of min^−1^), extravascular/extracellular volume fraction (*v*_*e*_), and *k*_*ep*_ (= *K*^*trans*^/*v*_*e*_, in units of min^−1^). The same Kety–Tofts analysis for computing* K*^*trans*^, *v*_*e*_, and *k*_*ep*_ in the tumor ROI was applied to extract quantitative information from a manually drawn ROI containing the temporalis muscle in each rat. The perfusion MRI parameters from muscle tissue served as a reference tissue to correct for variations in the tumor parameters between imaging visits (See Supplemental Methods for more details). Next, the DW-MRI data were analyzed using standard methods^12,35^ to arrive at the *ADC* for each voxel within the tumor ROI. The result of this analysis of multiparametric MRI data was a four-dimensional vector of MRI parameters, (*K*^*trans*^, *v*_*e*_* , k*_*ep*_, *ADC*), at each voxel within the tumor ROI of each animal at each imaging visit. All analyses were performed using MATLAB (Mathworks, Natick, MA).

### Identification of tumor habitats

All four-dimensional vectors of MRI parameters from all voxels within all tumor ROIs at all imaging visits were then pooled into one conglomerate feature matrix, where each column of the matrix represents one MRI parameter and each row corresponds to a voxel from a particular tumor ROI and imaging visit. Each column was normalized to the standard normal distribution in MATLAB to ensure that each MRI parameter had equal weight in the clustering algorithm for habitat identification^36^. Next, we followed a similar clustering procedure as described in previous work^7^, using *k*-means^37^ and agglomerative hierarchical clustering^38^ to identify three tumor habitats in terms of the level of vascularity (characterized by *K*^*trans*^ and *k*_*ep*_) and cellularity (characterized by *ADC* and *v*_*e*_) (See Supplemental Methods for further details). These habitats are defined as “high-vascularity low-cellularity” (HV-LC), “low-vascularity high-cellularity” (LV-HC), and “low-vascularity low-cellularity” (LV-LC), as established by Syed et al.^7^ At the end of the habitat identification process, all voxels within all tumor ROIs were assigned to one of the three habitats. The final result was a set of three time series for each rat: volumes (mm^3^) of HV-HC, LV-HC, and LV-LC as a function of time (days). Because no spatial information was included in the clustering, we performed multiregional spatial interaction (MSI) matrix analysis^39^ to verify that the identified habitats are spatially contiguous (see Supplemental Materials, Fig. S4, for complete details).

### The mathematical model family

**Figure 2:**
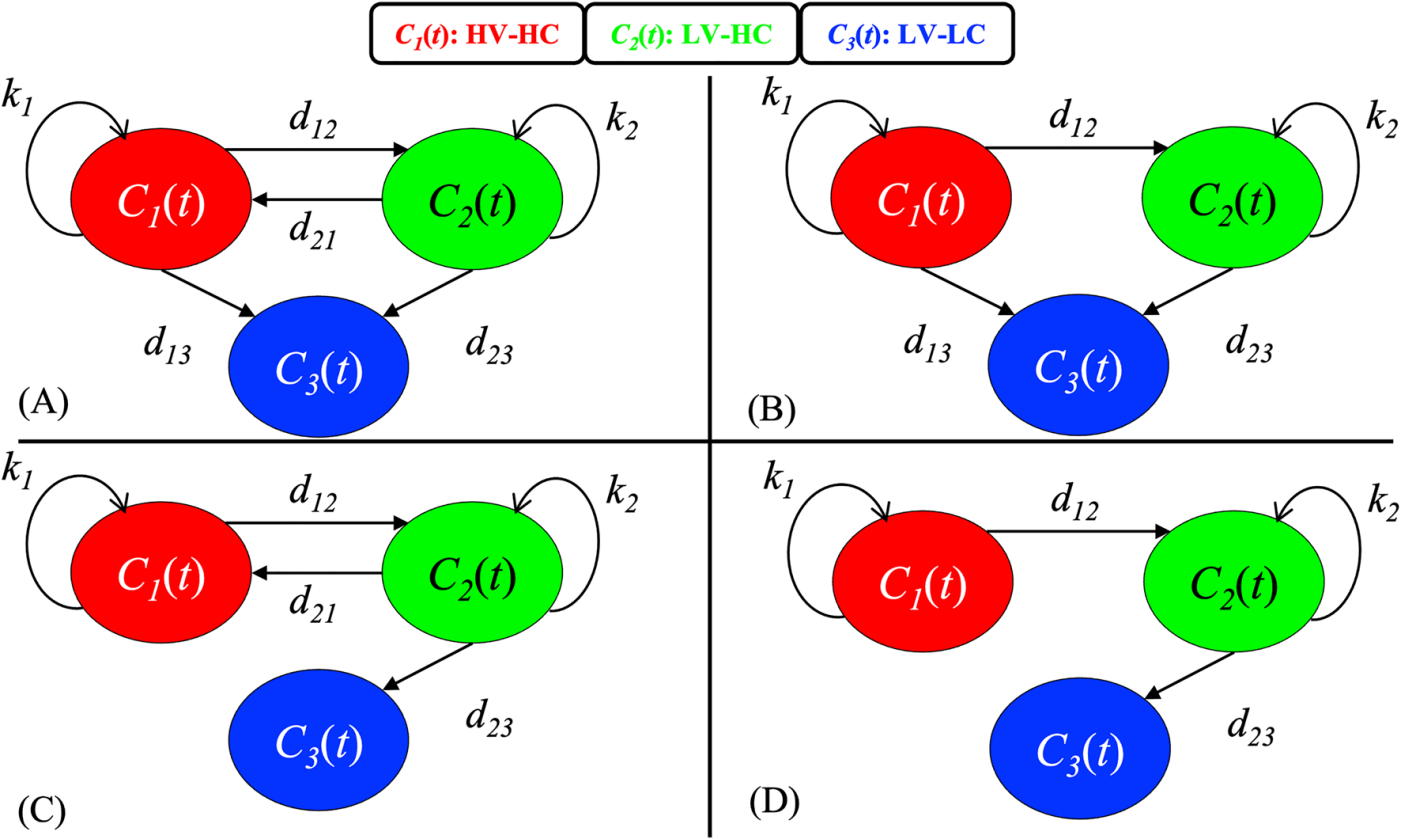
Visualizing a three-compartment mathematical model of tumor habitat growth. (**A**-**D**) correspond to Models (**A**-**D**), respectively, as introduced in Methods “The mathematical model family” section. In all panels, *C*_1_(*t*) corresponds to the HV-HC habitat (red), *C*_2_(*t*) corresponds to the LV-HC habitat (green), and *C*_3_(*t*) corresponds to the LV-LC habitat (blue). The parameters *k*_1_ and *k*_2_ are the growth rates of *C*_1_(*t*) and *C*_2_(*t*), respectively, while all parameters of the form *d*_*nm*__d__nm_ represent the transition rate from cluster *n* to cluster *m*. All model parameters are named and described in Table 1.

We developed a mathematical model of four equations to describe the dynamics of tumor habitats (Fig. 1B) identified in the eight control (C) rats and in eight selected rats from the two treated (R) cohorts (See Table S1 for the imaging schedule and radiation dose for these 16 rats). The subset of eight treated rats was selected from the cohort of 13 total treated rats based on uniformity of experimental details, such as the imaging schedule and the execution of the imaging protocol. From this initial set of four equations, we then develop a family of models with different underlying assumptions of model parameterizations (Fig. 2).

First, we introduce the parent model of four equations and seven parameters. We hypothesize that the HV-HC habitat will grow as the tumor recruits vasculature to its outer rim through angiogenesis^40^. As the tumor grows outward, former HV-HC voxels will become distanced from nearby vessels, leading to reduced perfusion and transition to the LV-HC habitat. There is a non-zero probability that *K*^*trans*^ may rise in LV-HC voxels as angiogenesis is triggered by hypoxia, leading to a transition from LV-HC to HV-HC membership. If LV-HC voxels do not succeed in recruiting vasculature, the LV-HC voxels will eventually become necrotic and irreversibly transition to LV-LC membership. For ease of writing the ODEs, we introduce the functions *C*_*1*_(*t*), *C*_*2*_(*t*), and *C*_*3*_(*t*) to represent the volumes (mm^3^) of the HV-HC, LV-HC, and LV-LC habitats, respectively, at time *t* (days). The parent model describing the hypothesized habitat interactions is then defined as follows:

1 $$V(t) = \frac{{C_{1} (t) + C_{2} (t) + C_{3} (t)}}{\theta }$$

2 $$\frac{{dC_{1} (t)}}{dt} = k_{1} C_{1} \left( t \right)\left( {1 - V\left( t \right)} \right) - d_{12} C_{1} \left( t \right) + d_{21} C_{2} (t) - d_{13} C_{1} (t)$$

3 $$\frac{{dC_{2} (t)}}{dt} = k_{2} C_{2} \left( t \right)\left( {1 - V\left( t \right)} \right) + d_{12} C_{1} (t) - d_{21} C_{2} (t) - d_{23} C_{2} (t)$$

4 $$\frac{{dC_{3} (t)}}{dt} = d_{13} C_{1} (t) + d_{23} C_{2} (t)$$

Equation (1) defines the total volume as a sum of the habitat volumes in relation to the carrying capacity, *θ*. This carrying capacity is left as a free parameter because the maximum size of the tumor that will lead to death may differ for each rat depending on a complex range of factors, such as location of the tumor, its vascularization, and its impact on healthy function^27,41^. The *k*_*n*_ and *d*_*nm*_ parameters refer to growth rates (mm^3^/day) of habitat *n* and decay rates (mm^3^/day) from habitat *n* to habitat *m*, respectively (Table 1 defines the model parameters).

**Table 1:** Parent model parameters and the bounds used in nonlinear fitting for model calibration. All growth rates, *k*_*n*_, and transition rates, *d*_*nm*_, are in units of mm^3^/day, and the carrying capacity, *θ*, is in units of *mm*^3^. The upper bound of the carrying capacity, *θ,* was chosen based on the known approximate volume of a rat’s brain. Upper bounds for all rates were chosen based on empirical growth rates of the tumors observed in the imaging data.

Parameters	Description	Lower bound	Upper bound
*k*_*1*_	Growth rate *C*_*1*_ (HV-HC)	0 mm^3^/day	5 mm^3^/day
*d*_*12*_	*C*_*1*_ → *C*_*2*_ transition rate	0 mm^3^/day	5 mm^3^/day
*d*_*21*_	*C*_*2*_ → *C*_*1*_ transition rate	0 mm^3^/day	5 mm^3^/day
*d*_*13*_	*C*_*1*_ → *C*_*3*_ transition rate	0 mm^3^/day	5 mm^3^/day
*k*_*2*_	Growth rate *C*_*2*_ (LV-HC)	0 mm^3^/day	5 mm^3^/day
*d*_*23*_	*C*_*2*_ → *C*_*3*_ transition rate	0 mm^3^/day	5 mm^3^/day
*θ*	Carrying capacity	0 mm^3^	2000 mm^3^

Equations (1)–(4) describe the parent model, termed Model A (Fig. 2A). Based on the results in “Results” section below, we were motivated to generate three additional models to generate a family of four total models: Model A, Model B (*d*_*21*_ = 0, Fig. 2B), Model C (*d*_*13*_ = 0, Fig. 2C), and Model D (*d*_*21*_ = *d*_*13*_ = 0, Fig. 2D). Model B describes the low probability of the LV-HC habitat transitioning to HV-HC, and Model C describes the low probability of the HV-HC habitat directly undergoing necrosis and transitioning to LV-LC membership. Model D combines the assumptions of Models B and C. 

### Model calibration to the habitat time series data

All four models were calibrated to the time series of tumor habitat volumes from each rat using the Levenberg–Marquardt method for nonlinear least-squares, implemented in MATLAB through the *lsqnonlin* function. MATLAB’s *multistart* function was used with *lsqnonlin* to evaluate 50 initial guesses and fixed lower and upper bounds (Table 1) for the model parameters of each model independently to obtain robust model fits and parameter values. The initial conditions for *C*_*1*_(*t*), *C*_*2*_(*t*), and *C*_*3*_(*t*) were defined by the volumes of the HV-HC, LV-HC, and LV-LC habitats, respectively, at the first imaging visit on day 10 post-inoculation. The time vectors of the datasets are redefined such that the initial images correspond to day 0 for easier comparison between plots.

### Model selection

The Akaike Information Criterion (*AIC*)^42^, corrected for small sample size, was used to select the most parsimonious model from the family of four models. The mathematical expression for the *AIC* is as follows:

5 $$\it \it \it {\text{AIC = Tln}}\left( {\frac{{{\text{RSS}}}}{{\text{T}}}} \right) + {\text{2k + }}\frac{{2{\text{k(k + 1)}}}}{{{\text{T}} - {\text{k}} - {\text{1}}}},$$

where *T* represents the total number of imaging visits, *k* is the total number of model parameters in the model, and the RSS is the root sum squared error. The four models were calibrated using the minimum number of imaging visits available (*T* = 5) across datasets, and the *AIC* was then computed for each model calibration for each rat. The model with the smallest *AIC* value was selected as the most parsimonious model.

### Prediction of tumor habitat dynamics

Two approaches were implemented to investigate the predictive ability of the model selected using the criterion introduced in “Model selection” section. The first approach is the leave-one-out (LOO) method in which we leave out the time series of the *j*th rat—serving as a validation dataset that we want to predict. We then compute the normalized weighted average of each model parameter distribution from the remaining seven rats in the population via three different weighting schemes: initial tumor volume, initial tumor habitat composition, and initial HV-HC volume (chosen due to having the smallest interquartile range in the treated cohort). The mathematical details of these weighting schemes are described in detail in the Supplemental Methods.

The second approach for evaluating the predictive ability of the selected model is bootstrapping^43^ (BS). Like the LOO approach, we take out rat *j* and compute the mean and standard deviation of the parameter distributions from the remaining rats in the population. Then, we generate a normal distribution for each parameter from the mean and standard deviation and sample this distribution *N*_*b*_ times between the 25th and 75th quartiles. We run the model forward for each set of sampled model parameters and arrive at *N*_*b*_ number of model curves, from which we compute the mean sampled curve and confidence intervals. This is the predicted fit for rat *j* when no habitat volumes beyond the initial visit are made available. As data becomes available, we update the model fit by taking a weighted average of the mean bootstrapped curve and rat *j*’s individually calibrated fit to the available data. We label this approach as bootstrapping with data updating. (See Supplemental Methods for mathematical details of both approaches).

### Statistical analysis

To determine significant differences in MRI parameter distributions between habitats, we employed the Kolmogorov–Smirnov two-sample test at a 5% significance level as well as a one-way analysis of variance (ANOVA). To evaluate the performance of each model during calibration, we introduced the normalized sum of square error (SSE), which is the sum of the square of the differences between the data and the model output for each habitat in each rat’s time series, normalized to the maximum volume of each habitat time series. The Wilcoxon rank sum test at a 5% significance level was used to evaluate significant differences in the SSE distributions of each habitat across the family of models to determine if any given model described a particular habitat with lower error. The concordance correlation coefficient (CCC)^44^ was also used to assess the agreement between the model outputs and the measured data, where CCC values above 0.8 are indicative of strong agreement between two distributions. We also use the rank sum test to determine which prediction method (i.e., LOO or bootstrapping) yielded the lowest error in predicting growth of the tumor habitats.

## Results

### Classification of image-informed tumor habitats

**Figure 3:**
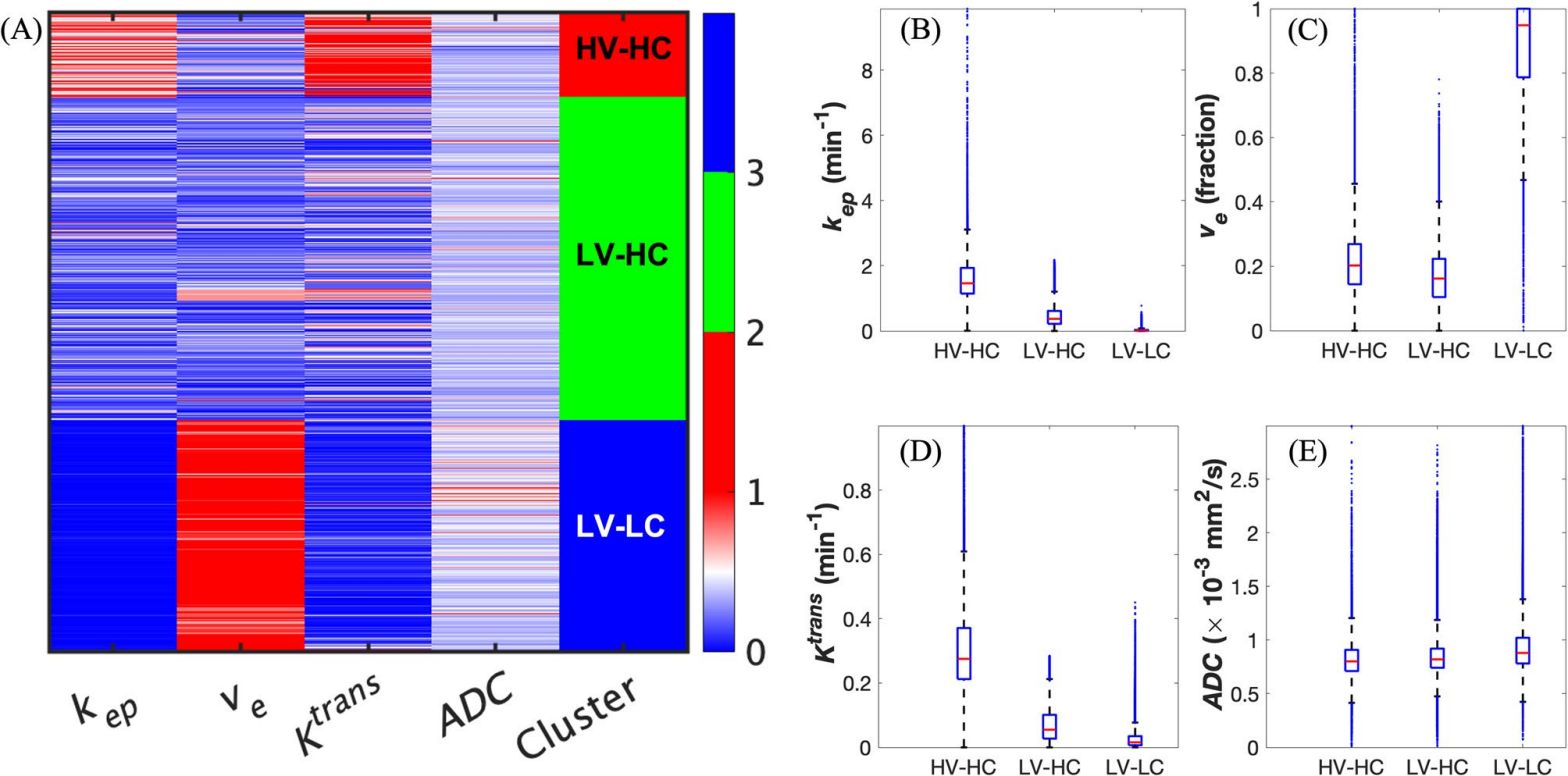
Identification of three tumor habitats with *k*-means clustering. (**A**) This is the heat map of normalized quantitative parameters grouped by the three identified clusters: high-vascularity high-cellularity (HV-HC), low-vascularity high-cellularity (LV-HC), and low-vascularity low-cellularity (LV-LC). (**B**) Boxplots of the distributions of *k*_*ep*_ are presented for each identified habit, where the red line in each boxplot represents the median of the distribution. Analogous to (**B**), panels (**C**-**E**) show the distributions of *v*_*e*_, *K*^*trans*^, and ADC, respectively. 168,207 voxels were included in this clustering analysis. We found that all four parameter distributions differ significantly between the three habitats, with *p* < 0.05.

Figure 1A provides an overview of the clustering pipeline, taking four MRI-informed parameter maps from each rat dataset and outputting three tumor habitats with distinct physiological characteristics, namely HV-HC, LV-HC, and LV-LC. We found that *k*-means clustering yielded habitats with greater separation between clusters, in terms of higher mean values of *K*^*trans*^ for the HC-HV clusters compared to the agglomerative clustering method. Moreover, qualitative inspection of the *k*-means clustering (Fig. 3A) and the agglomerative clustering outputs (Fig. S2A) shows that *k*-means collects stray voxels with high values of *K*^*trans*^ (colored in red) into the HV-HC habitat, whereas the agglomerative clustering method grouped these voxels into the LV-HC habitat, yielding higher mean vascularity (Fig. S2B, D) in the LV-HC habitat. Thus, we proceeded with the habitats identified by the *k*-means clustering algorithm for the rest of this study.

Physiological characteristics were determined by assessing the mean values of the MRI parameter distributions (Fig. 3B–E) within each habitat (Table S2). The mean values of *ADC* and *v*_*e*_ were used to assess cellularity, while the mean values of *K*^*trans*^ and *k*_*ep*_ were used to assess vascularity. The first habitat had a mean [*ADC*, *v*_*e*_] of [0.826 × 10^–3^ (± 0.216) mm^2^/s, 0.232 (± 0.158)] and mean [*K*^*trans*^, *k*_*ep*_] of [0.307 (± 0.155) min^−1^, 1.625 (± 0.788) min^−1^]. The second habitat had a mean [*ADC*, *v*_*e*_] of [0.852 × 10^–3^ (± 0.186) mm^2^/s, 0.181 (± 0.118)] and a lower mean [*K*^*trans*^, *k*_*ep*_] of [0.070 (± 0.054) min^−1^, 0.447 (± 0.310) min^−1^] compared to the first habitat. Finally, the third habitat had a substantially higher mean [*ADC*, *v*_*e*_] of [0.945 × 10^–3^ (± 0.280) mm^2^/s, 0.848 (± 0.199)] and significantly lower mean [*K*^*trans*^, *k*_*ep*_] of [0.0276 (± 0.034) min^−1^, 0.029 (± 0.097) min^−1^] compared to the other two habitats. All MRI parameter distributions were found to be significantly different between the three habitats with *p* < 0.0001 from all statistical tests. Thus, the first habitat (*C*_*1*_(*t*)) was labelled as HV-HC as it had the highest values of vascularity-related parameters (*K*^*trans*^ and *k*_*ep*_ report on vascularity) and the lowest values of cellularity-related parameters (*ADC* and *v*_*e*_ are inversely proportional to cellularity). The second habitat (*C*_*2*_(*t*)) was labelled as LV-HC as it was associated with lower *K*^*trans*^ and *k*_*ep*_ than the first habitat, and the third habitat (*C*_*3*_(*t*)) was labelled as LV-LC as it had the highest values of cellularity-related parameters and lowest measures of vascularity.

**Figure 4:**
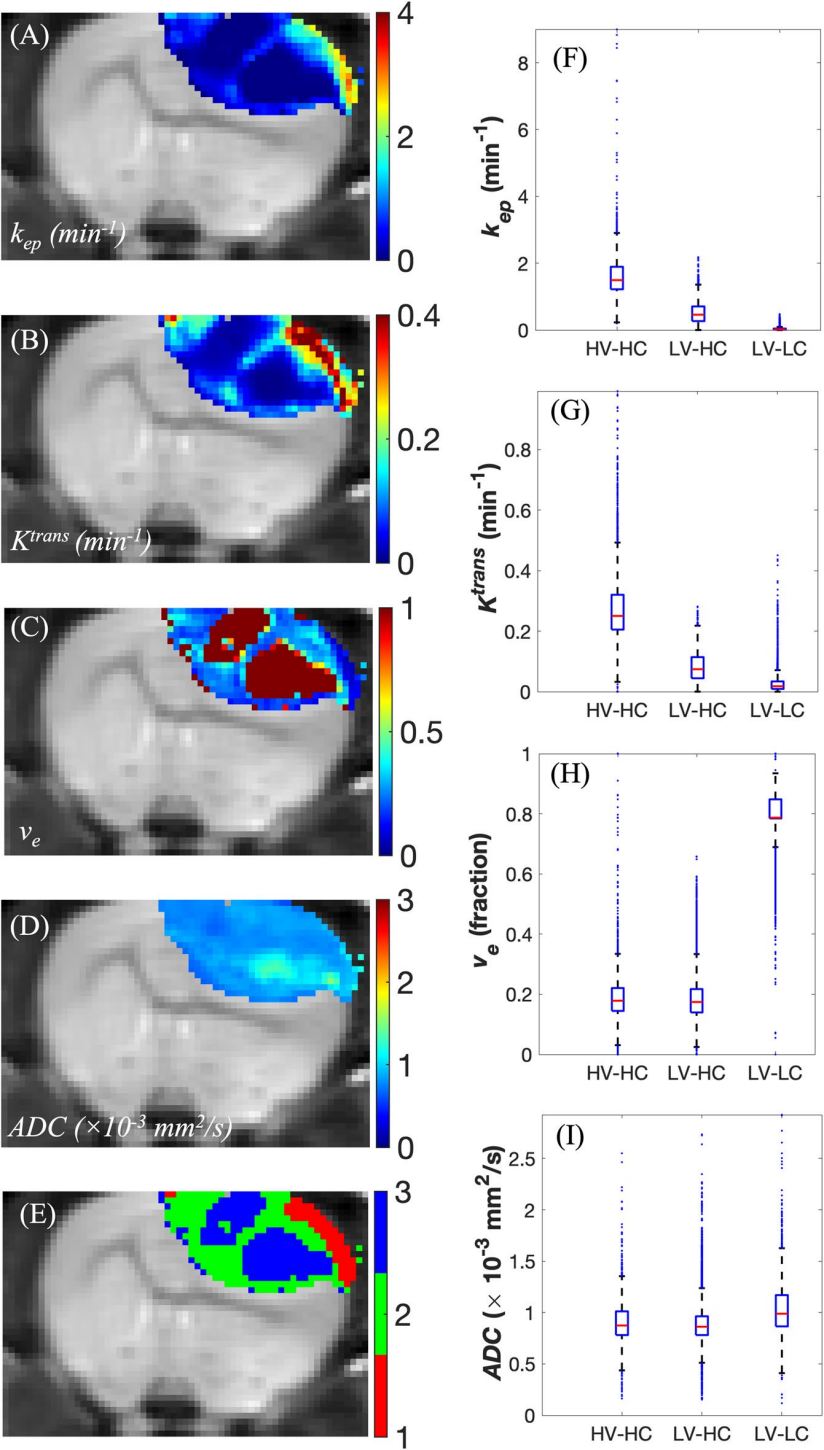
Quantitative parameter maps and tumor habitat map for a representative rat at the third imaging visit. (**A**-**D**) depict the *k*_*ep*_ (min^−1^), *v*_*e*_, *K*^*trans*^ (min^−1^), and ADC (10^−3^ s/mm^2^) maps, respectively. (**E**) This is the tumor habitat map where HV-HC is in red, LV-HC is in green, and LV-LC is in blue. The LV-LC cluster spatially localizes to the center of the tumor with high values of *v*_*e*_ and high values of the ADC, which align with expected observations in necrotic regions. The HV-HC habitat corresponds to the edge of the tumor, which exhibits characteristic high values of *K*^*trans*^ and *k*_*ep*_ most likely due to breakdown of the blood-brain-barrier. Lastly, the LV-HC region surrounds the necrotic regions, consisting of voxels with mid-range values of *K*^*trans*^ and lower values of *v*_*e*_ compared to the LV-LC habitat. (**F**-**I**) are the quantitative parameter distributions from each habitat, grouped by *k*_*ep*_, *K*^*trans*^, *v*_*e*_, and ADC, respectively. All four parameter distributions differ significantly between the three habitats (*p* < 0.05).

Figure 4 shows the MRI parameter maps (Fig. 4A–D) alongside the corresponding habitat map (Fig. 4E) for a representative rat imaged at visit 3. The distributions of *k*_*ep*_, *K*^*trans*^, *v*_*e*_, and *ADC* are displayed in Fig.  4F–I, with statistically significant differences between the parameter distributions of the three habitats (*p* < 0.001). Qualitatively, the habitats appear to be spatially contiguous. The highly perfused HV-HC habitat is at the well-vascularized edge of the tumor, while the LV-LC habitat overlaps with the necrotic center of the tumor. The LV-HC habitat spatially separates the other two habitats. The habitat maps for two representative rats from the control and treated cohorts, respectively, are displayed in Fig. S3 at multiple imaging visits. Quantitatively, MSI analysis (Fig. S4A-D) verifies that the habitats are spatially contiguous.

### Calibration of the family of mathematical models

The tumor habitat time series from all 16 rat datasets were used to calibrate each of the four mathematical models [Eqs. (1)–(4), Fig. 2]. The calibrated model parameters for Model A (the parent model) are presented in Table S3, and we found that the *d*_*21*_, *d*_*13*_, and *θ* parameter distributions are significantly different between the control (C) and treated (R) cohorts. Additionally, in Table S3, we observed that the model parameters *d*_*21*_ and *d*_*13*_ are one to two orders of magnitude smaller compared to the other model parameters, prompting the development of the additional three models that exclude these two model parameters.

**Figure 5:**
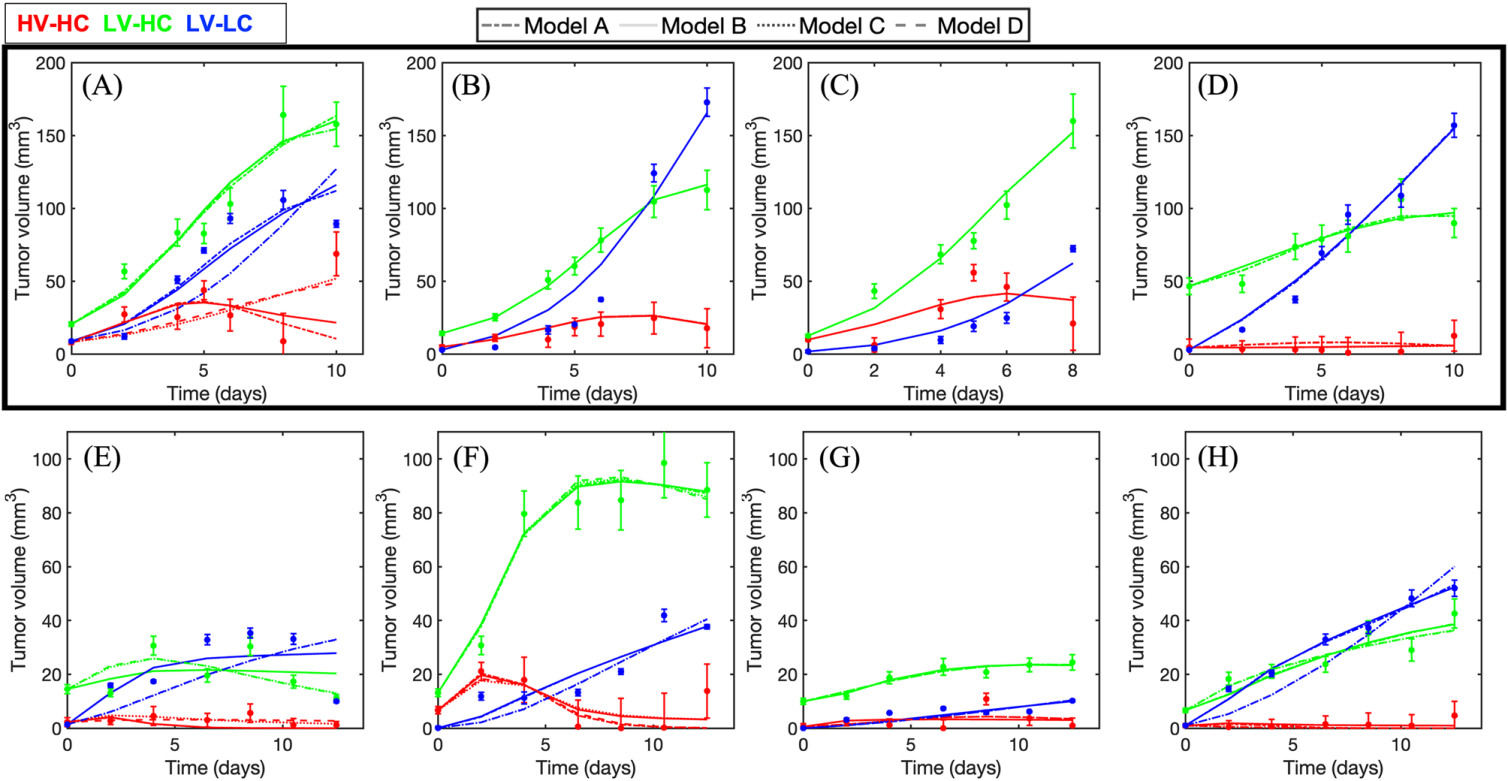
A family of four models calibrated to the habitat data. The first row (black rectangle) shows the results of the calibrations to four example control rats, while the second row shows analogous results for four example rats from the treated cohort (where (**E**-**G**) received 40 Gy of radiation and (**H**) received 20 Gy). Data are represented by solid circles with confidence intervals computed as described in the Supplemental Methods. Red, green, and blue correspond to the HV-HC, LV-HC, and LV-LC habitats, respectively. Each model is represented by a different line style as depicted in the legend at the top of the figure. There is high similarity between the fits of all four models across the datasets, suggesting that each model shows similar performance in describing the dynamics of each habitat time series and allowing for the selection of the most parsimonious model with the least number of free parameters. When comparing tumor sizes at the final imaging visit, the treated rats exhibited significantly smaller tumors than the control rats (*p* < 0.05). This observation is apparent as panels (**A**-**D**) have double the dynamic range of panels (**E**).

Panels A-D of Fig. 5 show all four models calibrated to the habitat times series of four representative rats from the control group, while panels E–H show these calibrations for four representative rats from the treated group (See Figs. S5 and S6 for all 8 control and all 8 treated rat calibrations, respectively). There were no statistical differences in the SSEs for all models when compared within identical habitats. Moreover, the four CCC values (Fig. S7) (one for each model) are all above 0.95, indicating strong agreement between the four models and the data that they were calibrated to.

### Selecting the most parsimonious model

The *AIC* values were computed using a MATLAB implementation of Eq. (5) for each of the four calibrated models across all 16 habitat time series, resulting in four AIC values per rat that are collected in Table S4. Model D consistently yielded the lowest *AIC* for all rat datasets, which is why Model D was selected as the most parsimonious model relative to the other three models (see Table S5 for model parameter values). Because the four models performed similarly (Figs. 5, S7), Model D was selected because it had the least number of parameters. Thus, Model D is used for the remaining analyses in this study.

### Prediction and validation of tumor habitat dynamics

Treating each tumor habitat time series independently as a validation dataset, we applied the LOO and bootstrapping methods to evaluate the ability of Model D to predict the habitat dynamics. To select the appropriate number of samples, *N*_*b*_, in bootstrapping, we performed the bootstrapping method for different values of *N*_*b*_, plotted the resultant SSEs against *N*_*b*_, and averaged the results across all rats in the control (Fig. S8A) and treated (Fig. S8B) cohorts. We qualitatively inspected the plots in Fig. S8 to arrive at *N*_*b*_ = 200 as an appropriate sample size at which the SSE values have stabilized and additional samples do not improve the prediction.

**Figure 6:**
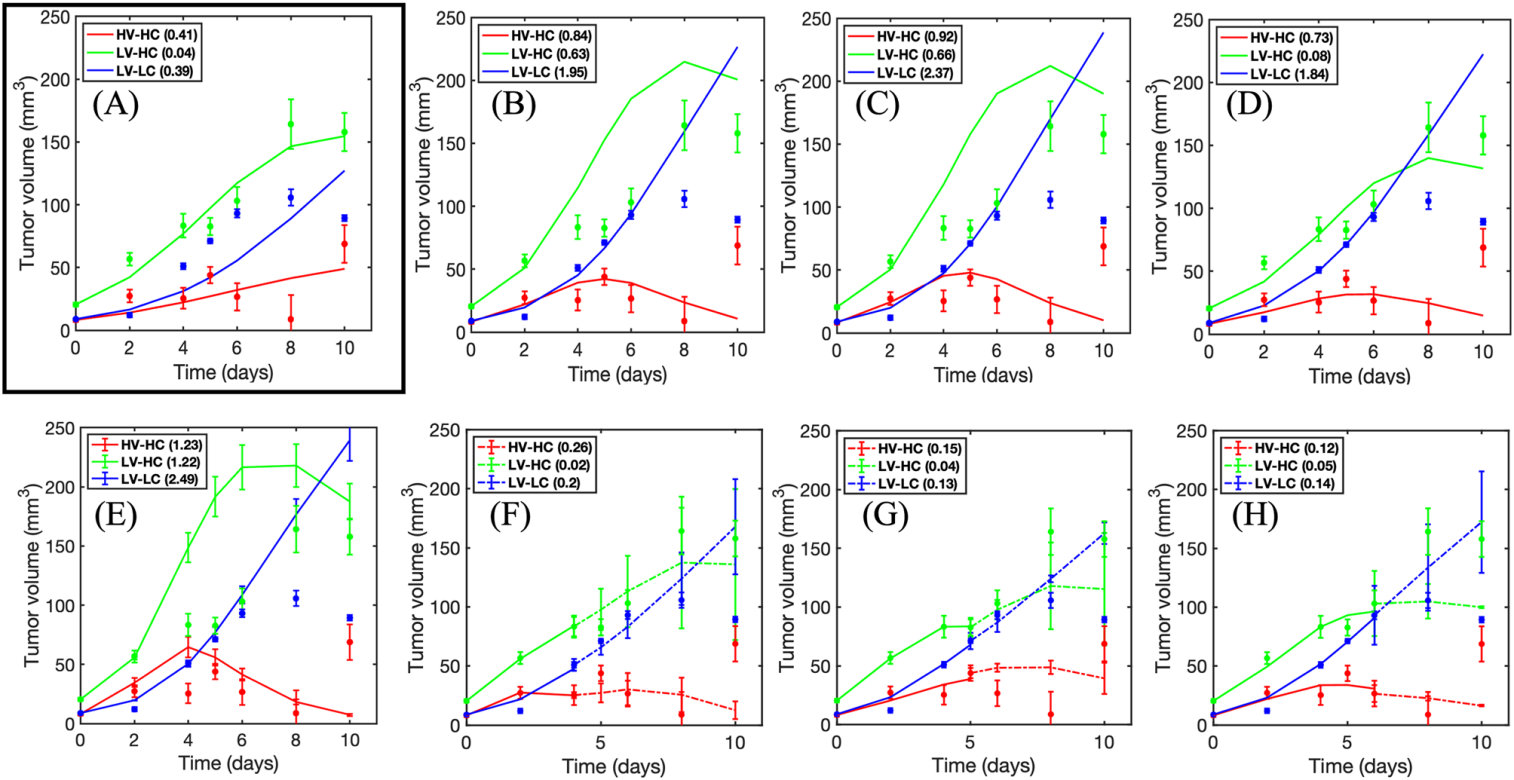
Predictions for a representative rat using the leave-one-out and bootstrapping approaches. (**A**) Model D calibrated to the habitat time series of a representative rat. The SSEs for each habitat are displayed in the legend. The black box emphasizes that this is the calibrated fit to which the predictions are to be compared. (**B**-**C**) are the predicted curves using the leave-one-out approach that weighs the prediction from the remaining population of rats by similarity in initial tumor volume, initial tumor composition, and initial HV-HC volume, respectively. (**E**) The bootstrapped prediction with the initial visit serving as an initial condition is plotted along with the measured habitat time series. (**F**–**H**) present bootstrapping with two, three, and four visits, respectively. The solid curves in (**F**–**H**) are the model calibration using the available data, whereas the dashed curves are the predictions forward. Bootstrapping, when incorporating additional imaging data to update the prediction, yields overall smaller SSEs than the leave-one-out approach.

**Figure 7:**
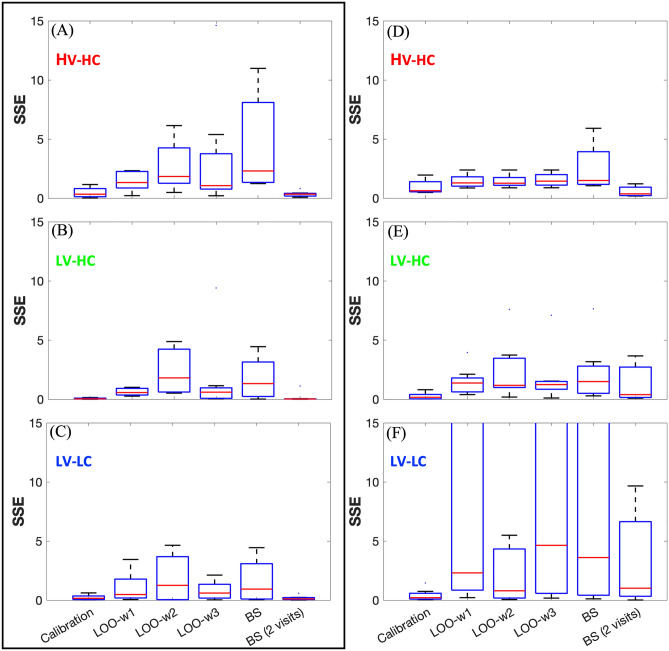
Comparing errors between predictions cross all rat datasets. (**A**-**C**) display the SSE distributions for six different prediction approaches for the HV-HC, LV-HC, and LV-LC habitats, respectively, for the control rat cohort (black rectangle). From left to right within each panel, the boxplots are model calibration, leave-one-out (LOO) with the initial tumor volume as the weighting scheme (LOO-w1), LOO with the initial tumor habitat composition as the weighting scheme (LOO-w2), LOO with the initial HV-HC volume as the weighting scheme (LOO-w3), bootstrapping (BS) with no data updating, and bootstrapping with two visits (BS (2 visits)). Bootstrapping with two visits yielded significantly lower error than the other four methods (*p* < 0.01) in the HV-HC and LV-HC habitat growth predictions. Furthermore, bootstrapping with two visits showed no significant differences in error when compared to the direct calibrations. (**D**-**F**) are the analogous results for the treated rat cohort. Similarly, bootstrapping with two visits yielded significantly lower SSEs compared to the remaining prediction methods in the HV-HC habitat growth predictions. SSEs in (**F**) were significantly higher than the SSEs in the remaining clusters, where the SSE distribution from the LOO-w2 had the lowest median and lowest interquartile range and outlier value. To provide an understanding of the anomalous ranges in panel (**F**), we report that the 75th percentile of LOO-w3 and BS did not exceed 30, whereas this value for LOO w1 was 53.06.

Figure 6 shows the results of the predictions for a representative rat. The calibrated model in Fig. 6A is juxtaposed with the LOO predictions (Fig. 6B–D) and the bootstrapping predictions (Fig. 6E–H). Using the initial tumor volume (Fig. 6B) and tumor composition (Fig. 6C) as weighting schemes in the LOO approach resulted in overestimation of the LV-LC and LV-HC habitat volumes at the final two imaging visits beyond the dynamic range of the data. Bootstrapping with data updating (Fig. 6F–H) decreased the error in the predictions at these later imaging visits. While the LOO predictions differ in accuracy depending on the weighting scheme, the predictions from bootstrapping with data updating yielded lower SSE values overall (Fig. 7). In the control cohort, the SSEs from the bootstrapping predictions were significantly lower (*p* < 0.05) than all SSEs from the LOO predictions for the HV-HC (Fig. 7A) and LV-HC (Fig. 7B) tumor habitats; this significance was also observed in the LV-LC LOO prediction using the weighting scheme determined by the initial tumor volume (Fig. 7C). Figure 7D–F shows the SSE distributions of the predictions for each habitat in the eight treated rats. The SSE values for bootstrapping with two visits in the data updating step were significantly lower (*p* < 0.05) than the remaining predictions for the HV-HC habitat alone. In all cases (Fig. 7A–F), bootstrapping with two visits yielded statistically similar SSE values compared to the individually calibrated fit.

## Discussion

To the best of our knowledge, we have presented the first application of an ODE-based model to describe the dynamics of image-informed tumor habitats. Because of the absence of spatial location in the ODE model, our proposed pipeline does not require accurately registered imaging data for habitat identification and subsequent mathematical modelling. This makes our pipeline amendable to fast analysis when image registration tools are not readily available or when the underlying anatomy makes registration challenging. Our method provides a global description of heterogeneous tumor growth—in the presence and absence of radiation therapy—and provides an avenue for predicting changes in heterogeneity and tumor progression.

The three identified habitats were determined based on variations in vascularity and cellularity and labelled accordingly as HV-HC, LV-HC, and LV-LC. Importantly, no spatial information was used during clustering, yet the habitats were found to spatially localize based on their similar properties, which was quantitatively confirmed with MSI analysis (Fig. S4). Spatial contiguousness is an important result based on the expected anatomy of growing solid tumors with a well-perfused rim and a necrotic core^45,46^. For example, across all rats, the HV-HC cluster was found at the outer rim of the tumor, while the LV-LC tumor was predominantly located at the center of the tumor (Fig. 4); and the LV-HC was found between the LV-LC and HV-HC habitats.

We employed model selection to identify the most parsimonious model for describing the tumor habitat dynamics. Model selection revealed that there are infrequent transitions from the LV-HC habitat to the HV-HC habitat as well as the HV-HC habitat directly to the LV-LC habitat (Table S3). Coupled with the observed localizations of each habitat at certain regions of the tumors, there is strong evidence that the LV-LC habitat correlates with necrosis typically found at the center of growing tumors. The LV-LC habitat exhibited significantly higher *ADC* values compared to the remaining two habitats, and high *ADC* values have been correlated with growth-induced and treatment-induced necrosis.^47,48^ The HV-HC cluster correlates with the highly vascularized rim in which perfusion parameters have been associated with biological markers of vascularization.^49,50^ Lastly, from previous work, the LV-HC region surrounding the necrotic tumor core has been associated with markers of hypoxia from reduced vasculature^7,51^. Interestingly, no “high-vascularity low-cellularity” (HV-LC) habitat was identified, likely because highly perfused regions result from angiogenesis by regions with sufficient cellular density, which direct nutrients to the tumor for further growth. 

The parameters defining the most parsimonious model as well as the above observations of the spatial localizations of the habitats suggest that, as the tumor grows, regions of HV-HC begin to lose access to vasculature and become regions of low vascularity while still maintaining cellularity (transitioning to the LV-HC habitat). As time elapses, we hypothesize that the cells within the LV-HC cluster begin to die from lack of oxygen formerly provided by the vasculature, leading to a decrease in cellularity and subsequent necrosis, which completes the transition to the LV-LC habitat. Prior work with more complex formulations of tumor region interactions have demonstrated the effect of hypoxic and necrotic regions in radiation therapy, where necrotic regions in particular have been shown to negatively affect treatment response^51,52^. The proliferation rates *k*_*1*_ and *k*_*2*_ may biologically represent the growth of cells under a nutrient rich environment and those under a nutrient poor environment, respectively. It would therefore be logical for *k*_*1*_ to be greater than *k*_*2*_. In our previous in vitro studies^53^ we observed the proliferation rate of C6 cells in a nutrient rich environment to be about 3.84 day^−1^, whereas in this study we observed an average *k*_*1*_ of 1.99 day^−1^ and maximum *k*_*1*_ of 4.89 day^−1^ for the most parsimonious model. While the in vitro estimate of *k*_*1*_ falls within the range of our in vivo estimated *k*_*1*_, we would expect these values to differ as there are inherent differences in in vivo and in vitro tumor growth as well as physiological heterogeneity between animals that influence the measured *k*_*1*_. 

Compared to other heterogeneity analyses and modelling methods, habitat imaging retains spatial information that is crucial for observations across time, like treatment response. Some therapies are known to affect functions of a tumor, such as vascularization, which would manifest as a relative shrinkage of the HV-HC habitat in our proposed analysis. Multi-species PDE model formulations from the literature have successfully linked the dynamics of hypoxic, necrotic, and perfused tumor regions in silico and in patient populations^29,30^, which demonstrate the potential to extend the complexity of our ODE formulation. The challenge, however, is in defining a complete mathematical description of the habitat interactions.

The most parsimonious ODE mathematical model in our formulation was successful in predicting global habitat dynamics, with the most successful method being bootstrapping with data updating. Bootstrapping with data updating yielded significantly lower error than the other prediction methods in the control cohort; however, the LV-LC habitat predictions in the treated cohort exhibited significantly higher error overall, even when using the parent model *in lieu* of the most parsimonious model. While this suggests that there are dynamics due to treatment that are not accurately captured, there is still utility in using the proposed ODE model for prediction. Radiation therapy confers marked decreases in vasculature^54,55^, manifesting as a decrease in the HV-HC habitat and an increase in the LV-HC habitat over time (Fig. S3), which is accurately captured by the model proposed in this study. When no imaging data is available beyond the initial visit, the LOO approach weighted by initial tumor volume or HV-HC volume performs better than bootstrapping alone as it directly considers measured biological information of other tumors in the population. Thus, there is flexibility in choosing a prediction approach based on data availability.

While the proposed pipeline for modelling tumor habitats confers some advances over existing modelling efforts for high grade glioma, there are opportunities for further investigation. First, while the identified habitats agree with previous work^7,8^ in which the habitats were histologically verified, there is a need to repeat histological analysis in this study of murine glioma. This added analysis would validate the image-based tumor habitats, making it feasible for future work to perform habitat imaging in independent cohorts of animals with greater certainty. Additionally, while the clustering analysis was done on the entire set of available rat data (control and treated), the mathematical modelling analysis was performed on a subset of the treated cohort along with the control cohort as explained in “The mathematical model family” section. An important next step is to repeat this analysis using a larger cohort of rats in each treatment group (20 or 40 Gy) to determine whether a modified ODE model is necessary to capture the effects of different therapy dosage regimens on the habitat dynamics.

## Conclusion

This study presented a pipeline for mathematically modelling image-derived tumor habitats in a murine model of glioma using a set of biology-based, coupled ordinary differential equations. We identified three distinct tumor habitats from clustering four sets of quantitative parameters computed from DCE- and DW-MRI data. All models in the family of ODE models exhibited errors that were not significantly different, allowing for the selection of the most parsimonious model that was parameterized by the least number of parameters. Model selection, coupled with observed habitat localizations, revealed important biological insights on glioma behavior that are corroborated by the literature. We found that the selected model could predict tumor dynamics. Further development and validation of this approach could yield accurate predictions of the dynamics of tumor physiology that may be leveraged to optimize therapy for individual tumors.

## Supplementary Information

Supplementary Information.

## Data Availability

The datasets generated during and/or analyzed during the current study are available from the corresponding author on reasonable request.
