# Author Correction: Mathematical modelling of the dynamics of image-informed tumor habitats in a murine model of glioma

**DOI:** 10.1038/s41598-023-35324-z

**Published:** 2023-05-22

**Authors:** Kalina P. Slavkova, Sahil H. Patel, Zachary Cacini, Anum S. Kazerouni, Andrea L. Gardner, Thomas E. Yankeelov, David A. Hormuth

**Affiliations:** 1 Department of Physics, The University of Texas at Austin, Austin, TX USA; 2 Department of Computer Science, Case Western Reserve University, Cleveland, OH USA; 3 Department of Bioengineering, University of Illinois, Urbana‑Champaign, IL USA; 4 Department of Radiology, The University of Washington, Seattle, WA USA; 5 Department of Biomedical Engineering, The University of Texas at Austin, Austin, USA; 6 Department of Diagnostic Medicine, The University of Texas at Austin, Austin, TX USA; 7 Department of Oncology, The University of Texas at Austin, Austin, TX USA; 8 The Oden Institute for Computational Engineering and Sciences, The University of Texas at Austin, 201 E 24Th Street, Austin, TX 78712 USA; 9 Livestrong Cancer Institutes, The University of Texas at Austin, Austin, TX USA; 10 Department of Imaging Physics, The University of Texas MD Anderson Cancer Center, Houston, TX USA

Correction to: *Scientific Reports* 10.1038/s41598-023-30010-6, published online 20 February 2023

The original version of this Article contained an error in the Funding section.

“We thank the National Institutes of Health for funding through NCI U01CA142565, U01CA174706, U01CA253540, U24CA226110, R01CA240589, and R01CA260003, as well as NIH U24EB029240.”

now reads:

“We thank the National Institutes of Health for funding through NCI U01CA142565, U01CA174706, U01CA253540, U24CA226110, R01CA240589, and R01CA260003.”

The original Article has been corrected.

