# Supplementary material for: Mathematical modelling of the dynamics of image-informed tumor habitats in a murine model of glioma
Source: Sci Rep. 2023 Feb 20;13:2916. doi: 10.1038/s41598-023-30010-6 (PMC9941120; doi:10.1038/s41598-023-30010-6)
Supplement: Supplementary file 1 — Supplementary Information. [file 41598_2023_30010_MOESM1_ESM.docx]

***Supplemental Materials*
Mathematical modelling of the dynamics of image-informed tumor habitats in a murine model of glioma**

Kalina P. Slavkova, Sahil H. Patel, Zackary Cacini, Anum S. Kazerouni, Andrea Gardner, Thomas E. Yankeelov, David A. Hormuth, II

Supplemental Methods

*Animal model and magnetic resonance imaging*

As stated in the main text, all experimental procedures were approved by our Institutional Animal Care and Use Committee and were performed in accordance with relevant guidelines and regulations. This study is reported in accordance with ARRIVE guidelines (https://arriveguidelines.org/). Female Wistar rats (N = 21) were anesthetized with 2% isoflurane in 98% oxygen for all imaging and surgical procedures, where body temperature was maintained near 37^o^C by a flow of warm air, and the respiration rate was monitored using a pneumatic pillow. Eight days after the glioma cells were implanted, a jugular catheter was placed within each rat for injection of a gadolinium-based contrast agent (Gado-DTPA^TM^, BioPal, Worcester, MA, USA). Rats were imaged beginning 10 days post-inoculation and then every 1-2 days after that for a total of 5-7 MRI data sets per animal. DW-MRI and DCE-MRI were collected on a 9.4T horizontal-bore magnet (Agilent, Santa Clara, CA, USA) using a 38 mm diameter Litz quadrature coil (Doty Scientific, Columbia, SC, USA). MR images were acquired over a 32 × 32 × 16 mm^3^ field of view sampled with a 128 × 128 × 16 matrix. A mutual information-based rigid registration algorithm ^1,2^ was used at the scanner at the beginning of the second through final imaging session to register the current imaging volume to the initial imaging volume (on day 10). Rats in the two treatment groups received a single dose of radiation therapy between the third and fourth imaging visits.

For the DW-MRI experiment, data was acquired using a pulsed fast spin-echo diffusion sequence with three orthogonal diffusion encoding directions with *b*-values of 150, 500, and 1000 s/mm^2^, *Δ*/*δ* = 25 ms/2 ms, and 16 averaged excitations. For the DCE-MRI experiment, we first collected a pre-contrast *T_1_* map using an inversion-recovery snapshot sequence with *TR*/*TE* = 5000/3 ms, 8 *TI*s (inversion times) logarithmically spaced between 200 - 4000 ms, and two averaged excitations. A series of *T_1_*-weighted images were then collected using a spoiled gradient echo sequence with *TR*/*TE* = 45/1.4 ms, two averaged excitations, a flip angle = 20°, and a temporal resolution of ~8.8 ms. This series of images were collected before, during, and for up to 20 minutes after the injection of a 200 µL bolus (0.05 mmol kg^-1^) of contrast agent through the jugular catheter. The contrast agent bolus was delivered over 5 seconds using a power injector.

*Computing multiparametric quantitative data*

In fitting the Kety-Tofts model to the DCE-MRI data as described in the main text, a rat population arterial input function (AIF) ^3^ was used as an input into the Kety-Tofts model, where, for each animal and imaging visit, the population AIF was scaled so that the concentration time course of a 20-voxel region of interest within the temporalis muscle yielded a *v_e_* of 0.11 ^4,5^. All values of *v_e_* > 1.0 were set to 1.0, and the remaining parameters were filtered within a physiological range such that 0.0 < *v_e_* < 1.0, 0.0 < *K^trans^* < 3.0 min^-1^, and 0.0 < *k_ep_* < 10.0 min^-1^, and a scale correction was applied using the same parameters computed in the muscle tissue (see next section for scale correction details). Next, the DW-MRI data were analyzed using standard methods^6,7^ to arrive at the *ADC* for each voxel within the tumor ROI; all ADC values were filtered such that 0.0 < *ADC* < 1.0×10^-3^ mm^2^/s.

*Scale correction of perfusion parameters in the tumor*

The same Kety-Tofts analysis for computing *K^trans^*, *v_e_*, and *k_ep_* described in Section 2.2 of the Methods in the main body text was applied to extracting quantitative information from a manually drawn ROI containing the temporalis muscle in each rat. The multiparametric data from muscle tissue served as a reference tissue to correct for variation in quantitative perfusion parameters between imaging visits.^8–10^ Specifically, we excluded the *j^th^* rat from the cohort of 21 rats and computed the median of the Kety-Tofts muscle parameter distributions combined from the 20 remaining animals (See Figure S1 for an example scaling). The scale factors were formed by dividing the resulting medians of each quantitative muscle parameter by the median of each quantitative muscle parameter from each visit in rat *j*’s dataset. We then scaled the tumor parameter distributions of rat *j* at each visit by these resulting scale factors. This procedure was repeated for all rats.

*Application of clustering algorithms for identifying tumor habitats*

Two clustering algorithms with *k* = 3 (from the literature^11,12^) were applied to the matrix; namely *k*-means clustering^13^ and agglomerative hierarchical clustering^14^ using Ward’s linkage as the distance metric. The spatial locations of the voxels within the ROI were not provided to the clustering algorithms. For each identified cluster – now referred to as a habitat – from both clustering algorithms, we calculated the mean value of each of the four quantitative parameters to determine the physiology of the habitat.

To estimate confidence intervals for the habitat time series, the clustering analysis was repeated with four groups of fifty instances of uniformly distributed random noise that were created based on repeatability studies of DW-MRI^15^ and DCE-MRI^6,10^ that reported the levels of noise in *K^trans^*, *v_e_*, *k_ep_*, and *ADC*. Each group of fifty instances was added to the corresponding MRI parameter distribution, and the noise-injected parameters were then passed through the clustering algorithms. Finally, the mean and confidence intervals were computed from the fifty clustering outputs.

*Leave-one-out approach for model prediction*

We consider a feature vector, $\vec{\text{f}}$, for computing the normalized weights, $\vec{w}$, in the weighting schemes. To establish the three weighting schemes, we consider three different features that $\vec{\text{f}}$ may represent: initial tumor volume, initial tumor habitat composition, and initial HV-HC volume (chosen due to having the smallest interquartile range in the treated cohort). In each of these weighting schemes, we seek to weigh more heavily the rats in the population that have a value of a given feature that is similar to the value of that feature in rat *j*. First, the non-normalized weights are computed as ${\hat{\text{w}}}_{\text{i}}\text{=}\left( \text{f}_{\text{j}}\text{-}\text{f}_{\text{i}} \right)^{\text{-1}}$, where $f_{j}$ is the feature (i.e., initial tumor volume, habitat composition, or HV-HC volume) of rat *j*, and $f_{i}$ is that same feature for rat *i* from the population. Finally, each normalized element in $\vec{w}$ is computed as

|  | $\text{w}_{\text{i}}\text{=}{\hat{w}_{i}}/{\sum_{\text{k≠j}}^{\text{N-1}} \hat{w}_{k}}$, | (1) |
| --- | --- | --- |

Once the weighted average of model parameters is computed, the model is run forward with these average parameters as input, yielding the prediction for the tumor habitat dynamics of rat *j*. This is then repeated for all rat datasets.

*Bootstrapping with data updating for model prediction*

As data becomes available, we update the model fit to by taking a weighted average of the mean model curve and rat *j*’s individually calibrated fit to the available data. The weights in this weighted average are defined as $\vec{\text{w}}\text{ =}\left[ \text{1-}\frac{\text{s}}{\text{T}}\text{,}\frac{\text{s}}{\text{T}} \right]$, where *s* is the number of data points currently available, and *T* is the total number of data points possible (number of imaging visits). The first element in $\vec{\text{w}}$ weights the mean sampled curve, and the second element weights the individually calibrated fit. For example, bootstrapping with three visits refers to the population-averaged bootstrapping prediction averaged with the fit to three imaging visits beyond the initial visit for the individual.

*Supplemental Methods References*

1. Colvin, D. C. *et al.* Earlier detection of tumor treatment response using magnetic resonance diffusion imaging with oscillating gradients. *Magn Reson Imaging* **29**, 315–23 (2011).

2. Hormuth II, D. A. *et al.* Predicting in vivo glioma growth with the reaction diffusion equation constrained by quantitative magnetic resonance imaging data. *Phys Biol* **12**, 46006 (2015).

3. Hormuth II, D. A., Skinner, J. T., Does, M. D. & Yankeelov, T. E. A comparison of individual and population-derived vascular input functions for quantitative DCE-MRI in rats. *Magn Reson Imaging* **32**, 397–401 (2014).

4. Donahue, K. M. *et al.* Dynamic Gd-DTPA enhanced MRI measurement of tissue cell volume fraction. *Magn Reson Med* **34**, 423–32 (1995).

5. Li, X. *et al.* Dynamic-contrast-enhanced-MRI with extravasating contrast reagent: rat cerebral glioma blood volume determination. *Journal of Magnetic Resonance* **206**, 190–9 (2010).

6. Barnes, S. L., Whisenant, J. G., Loveless, M. E., Ayers, G. D. & Yankeelov, T. E. Assessing the reproducibility of dynamic contrast enhanced magnetic resonance imaging in a murine model of breast cancer. *Magn Reson Med* **69**, 1721–1734 (2013).

7. Arlinghaus, L. R. & Yankeelov, T. E. Diffusion-Weighted MRI. in *Quantitative MRI in Cancer; Imaging in Medical Diagnosis and Therapy* (eds. Yankeelov, T. E., Pickens, D. R. & Price, R. R.) 91–107 (CRC Press, 2011).

8. Galbraith, S. M. *et al.* Reproducibility of dynamic contrast-enhanced MRI in human muscle and tumours: comparison of quantitative and semi-quantitative analysis. *NMR Biomed* **15**, 132–142 (2002).

9. Yankeelov, T. E. *et al.* Quantitative pharmacokinetic analysis of DCE-MRI data without an arterial input function: A reference region model. *Magn Reson Imaging* **23**, 519–529 (2005).

10. Barnes, S. L., Whisenant, J. G., Loveless, M. E. & Yankeelov, T. E. Practical Dynamic Contrast Enhanced MRI in Small Animal Models of Cancer: Data Acquisition, Data Analysis, and Interpretation. *Pharmaceutics* **4**, 442–478 (2012).

11. Kazerouni, A. S. *et al.* Quantifying Tumor Heterogeneity via MRI Habitats to Characterize Microenvironmental Alterations in HER2+ Breast Cancer. *Cancers (Basel)* **14**, 1837 (2022).

12. Syed, A. K., Whisenant, J. G., Barnes, S. L., Sorace, A. G. & Yankeelov, T. E. Multiparametric analysis of longitudinal quantitative MRI data to identify distinct tumor habitats in preclinical models of breast cancer. *Cancers (Basel)* **12**, 1–20 (2020).

13. Macqueen, J. *SOME METHODS FOR CLASSIFICATION AND ANALYSIS OF MULTIVARIATE OBSERVATIONS*.

14. Nielsen, F. Heirarchical clustering. in *Introduction to HPC with MPI for Data Science; Undergraduate Topics in Computer Science* 195–211 (Springer International Publishing, 2016).

15. Whisenant, J. G. *et al.* Assessing reproducibility of diffusion-weighted magnetic resonance imaging studies in a murine model of HER2+ breast cancer. *Magn Reson Imaging* **32**, 245–249 (2014).

Supplemental Figures


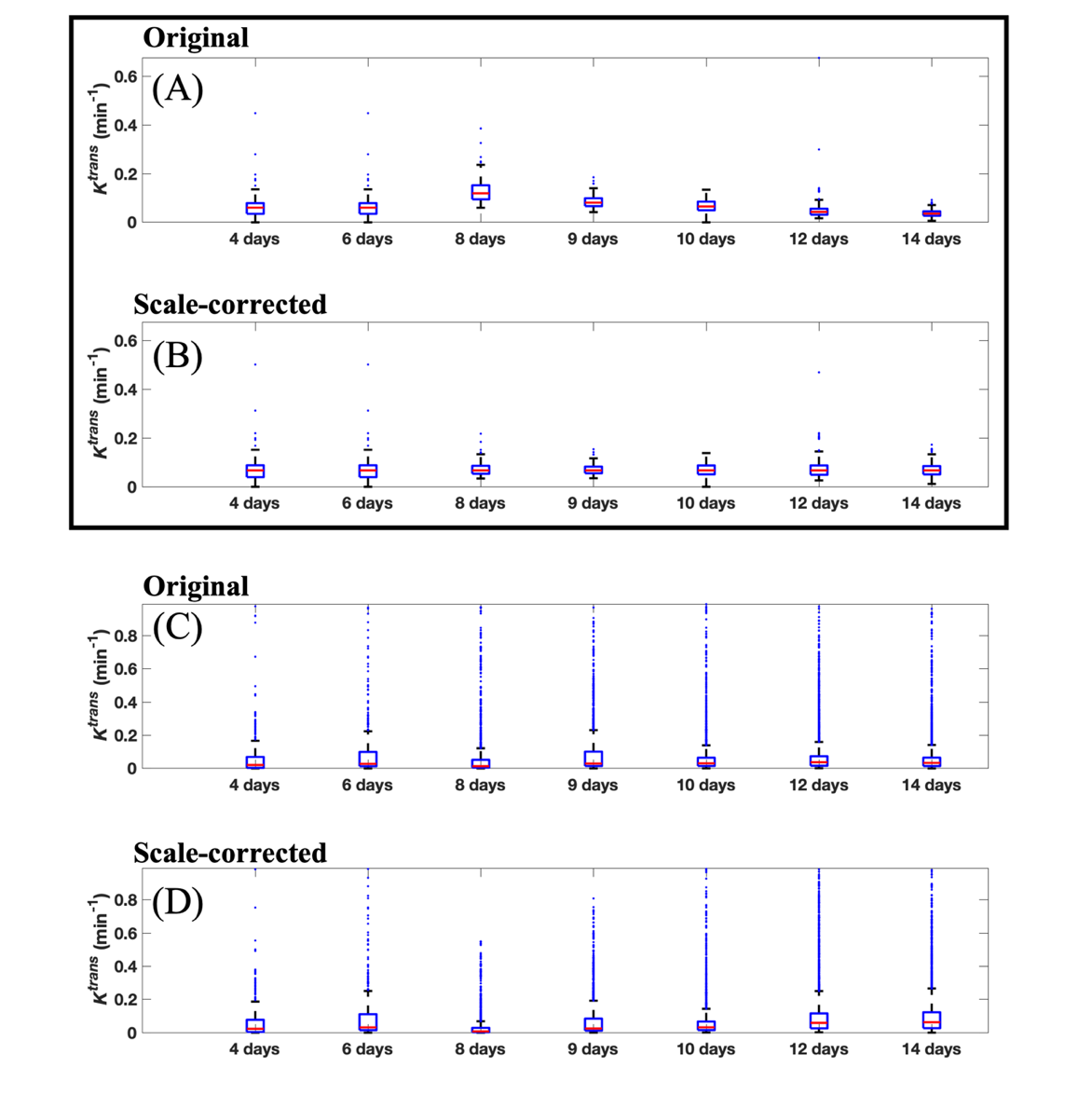


**Figure S1. Parameter distributions from the muscle and tumor of a representative rat.** (A) Distribution of *K^trans^* (min^-1^) in the temporalis muscle at all imaging visits. (B) Distribution of *K^trans^* in the temporalis muscle at all imaging visits after scaling to the median of the muscle *K^trans^* distribution across all rats. Scale-correction serves to minimize variation in the muscle parameters across imaging visits, which subsequently minimizes variation in tumor parameters that arise from system noise. (C) Analogous to panel (A) but for the tumor rather than the temporalis muscle, where the tumor *K^trans^* distribution was scaled by the median of the muscle *K^trans^* distribution. (D) Analogous to panel (B) but for the tumor.


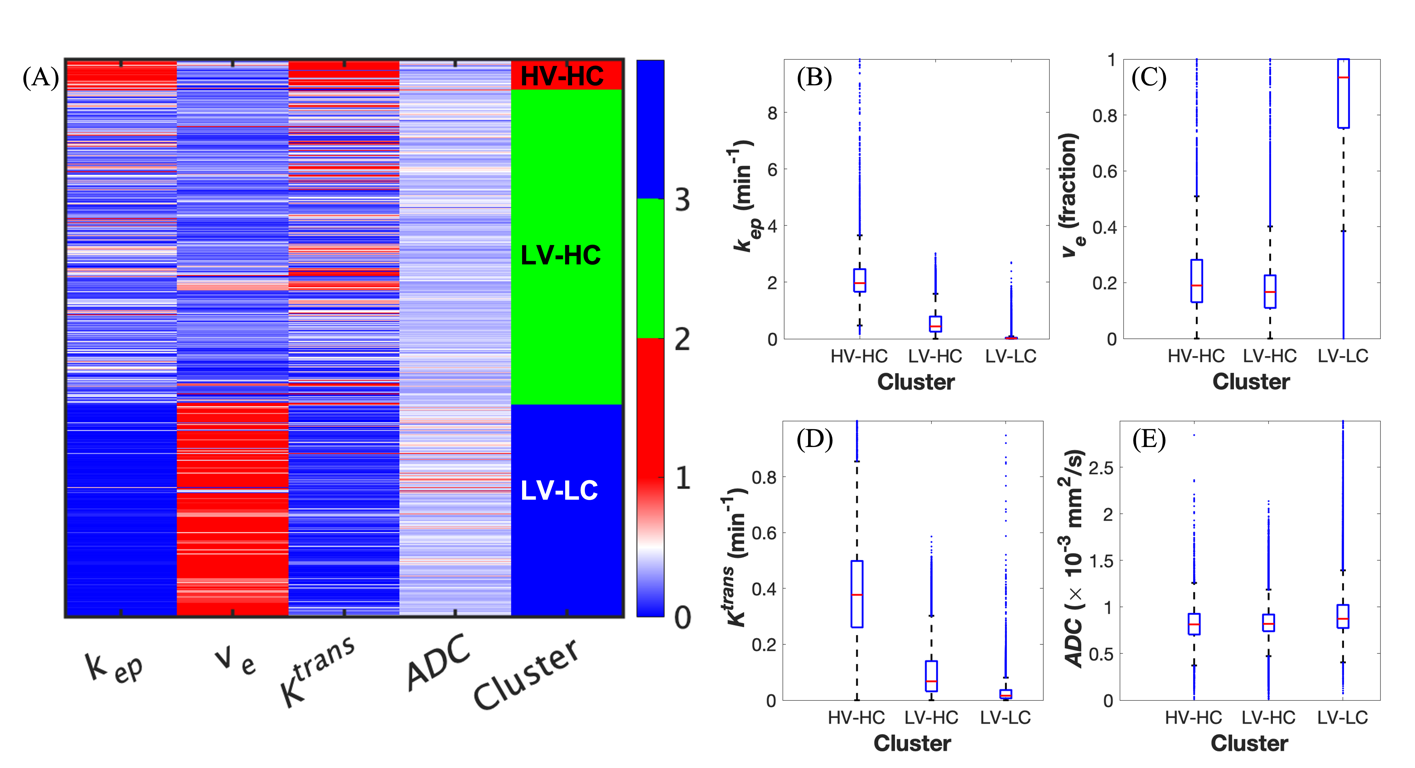


**Figure S2: Identification of three tumor habitats with agglomerative clustering**. (A) Heat map of normalized quantitative parameters grouped by the three identified clusters: high-vascularity high-cellularity (HV-HC), low-vascularity high-cellularity (LV-HC), and low-vascularity low-cellularity (LV-LC). (B) Boxplots of the distributions of *k_ep_* in each identified habit, where the red line in each boxplot represents the median of the distribution. The means of each habitat, from left to right, are 2.140 (±0.918), 0.565 (±0.421), and 0.040 (±0.097). (C) Distributions of *v_e_* with habitat means of 0.230 (±0.181), 0.188 (±0.134), and 0.848 (±0.199). (D) Distributions of *K^trans^* with habitat means of 0.387 (±0.197), 0.096 (±0.085), and 0.028 (±0.034). (E) Distributions of ADC with habitat means of 0.816 (±0.207), 0.843 (±0.166), and 0.945 (±0.279). We found that all habitats exhibit statistically significant difference in their four quantitative parameter distributions, where *p* <0.05.


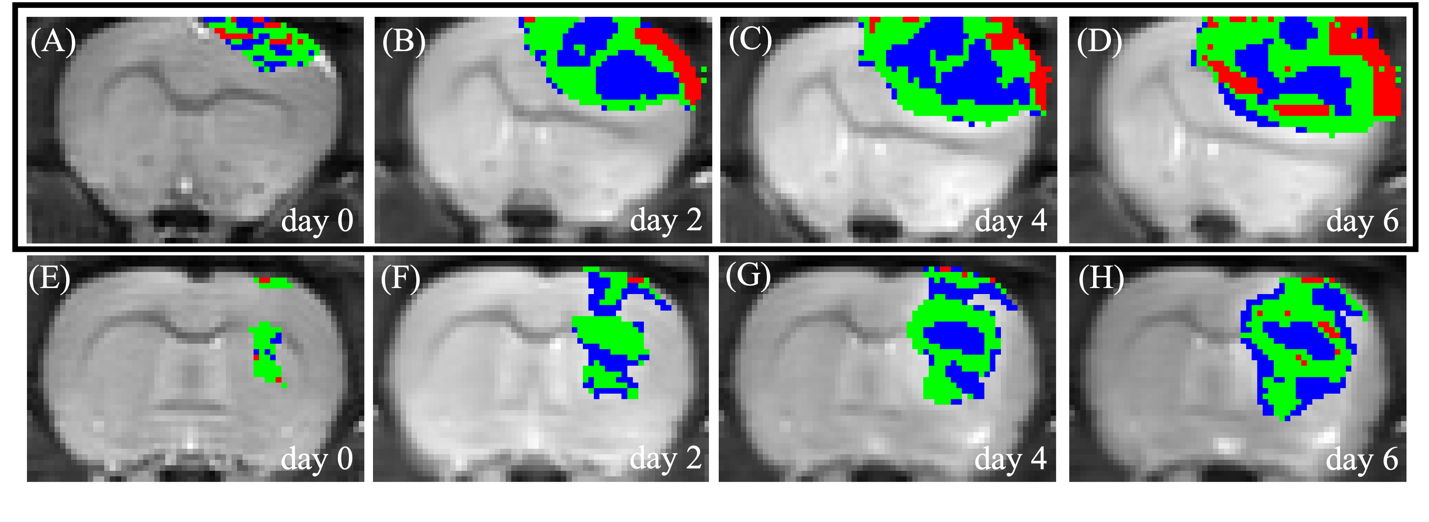


**Figure S3: Habitat maps for representative rats from the control and treated cohorts**. (A)-(D) Habitat maps from a representative control rat for day 0 through day 6, where day 0 indicates the initial imaging visit collected at 10 days post-inoculation of glioma cells into the animal. The HV-HC habitat is in red, the LV-HC habitat is in green, and the LV-LC habitat is in blue. (E)-(H) Habitat maps from a representative treated rat for the same span of imaging visits. Control tumors exhibit a larger proportion of the HV-HC habitat compared to treated tumors.


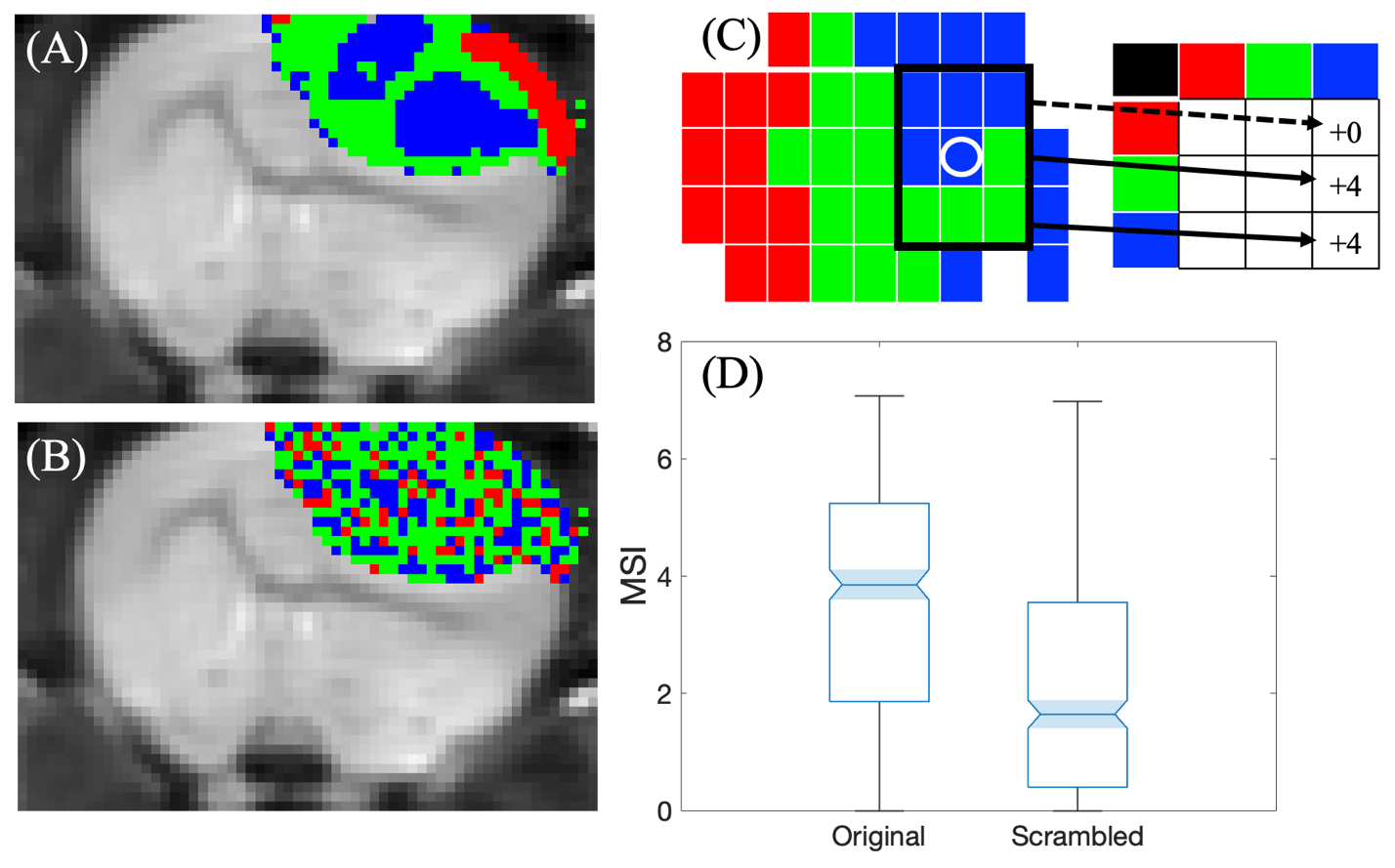


**Figure S4: Multiregional spatial interaction (MSI) to quantify habitat contiguousness**. (A) Habitat maps for all imaged rat tumors were computed by applying *k*-means clustering to a conglomerate matrix of quantitative MRI data across all tumor voxels. Voxel locations were excluded from the *k*-means analysis. (A) Representative tumor with original habitat mapping outputted from the *k*-means clustering analysis. (B) Scrambled habitat map where the voxels in the original habitat map were randomly permuted to new locations within the tumor. For this representative rat, the MSI matrix was computed by counting the neighbors of each voxel that belonged to each habitat. (C) The black box encloses the neighbors of the pixel for which the neighbor interactions are being analyzed (white circle). This pixel has four blue neighbors (LV-LC), four green neighbors (LV-HC), and no red neighbors (HV-HC). Thus, these counts can be added to the cells in the last column of the MSI matrix, which holds the neighbor counts for all LV-LC pixels within the tumor. The black box can be slid to center a new pixel within the tumor, and the process is repeated until all neighbor counts for all pixels are totaled. Because the in-plane resolution was smaller than the through-plane resolution, the MSI matrix was computed for each 2D slice of the imaged tumor volume; and the values along the diagonal of the final MSI matrix are normalized by the respective habitat volume. This MSI analysis process is repeated for all 21 rats. (D) Comparing the MSI distributions between the original and scrambled habitat maps for all rats. The MSI was significantly higher (*p*<0.05) for the original mapping compared to the scrambled mapping, indicating that the habitats are spatially contiguous.


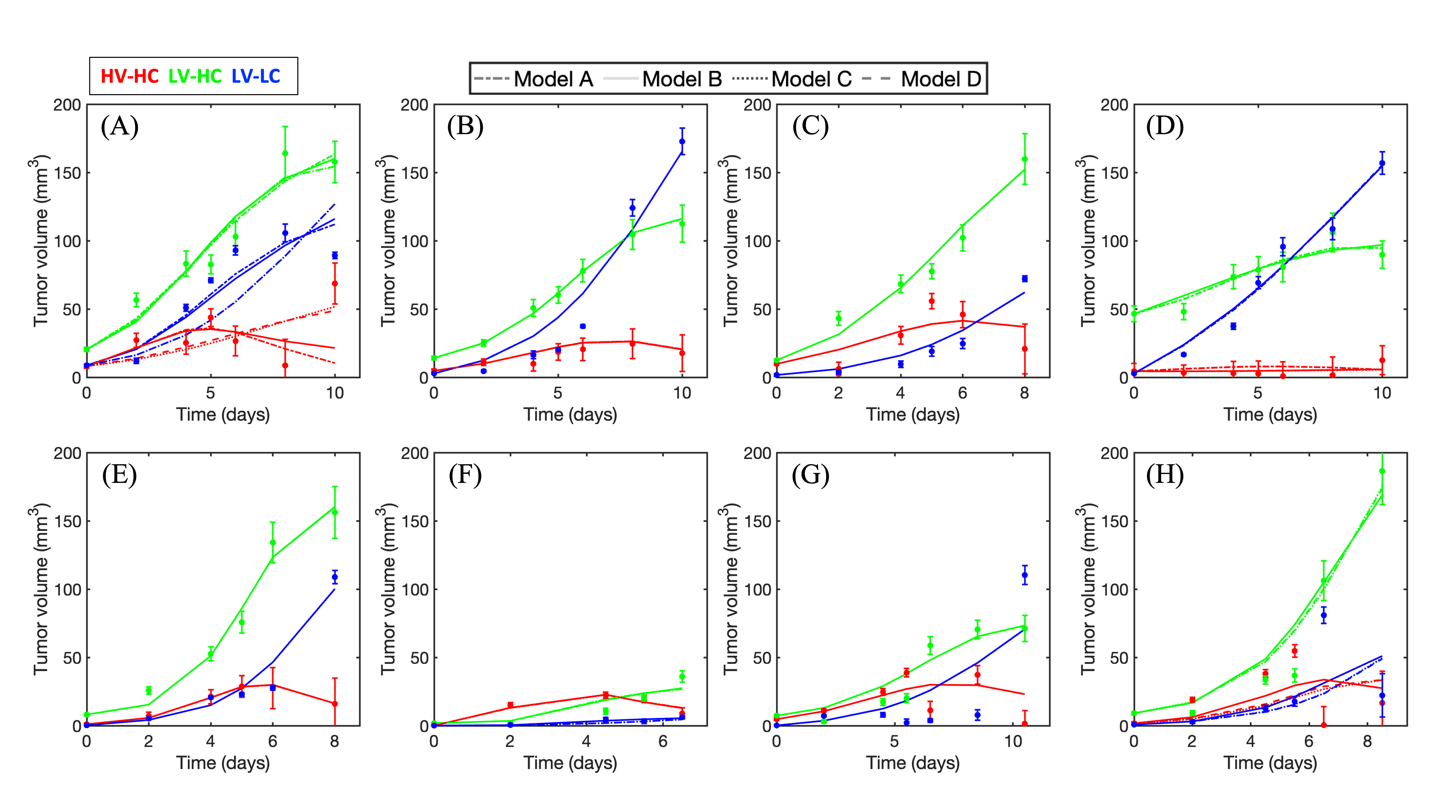


**Figure S5: A family of four models calibrated to the habitat time series of all eight control rats.** Each panel displays the model calibrations for a particular rat dataset from the control group that received no treatment (See Table S1 for each rat’s imaging schedule, where (A) is rat C1, (B) is rat C2, and so on). Data are represented by solid circles with confidence intervals computed as described in the Methods. Red, green, and blue correspond to the HV-HC, LV-HC, and LV-LC habitats, respectively. Each model is represented by a different line style as depicted in the legend at the top of the figure. There is high similarity between the fits of all four models across all rats, suggesting that each model shows similar performance in describing the dynamics of each habitat time series.


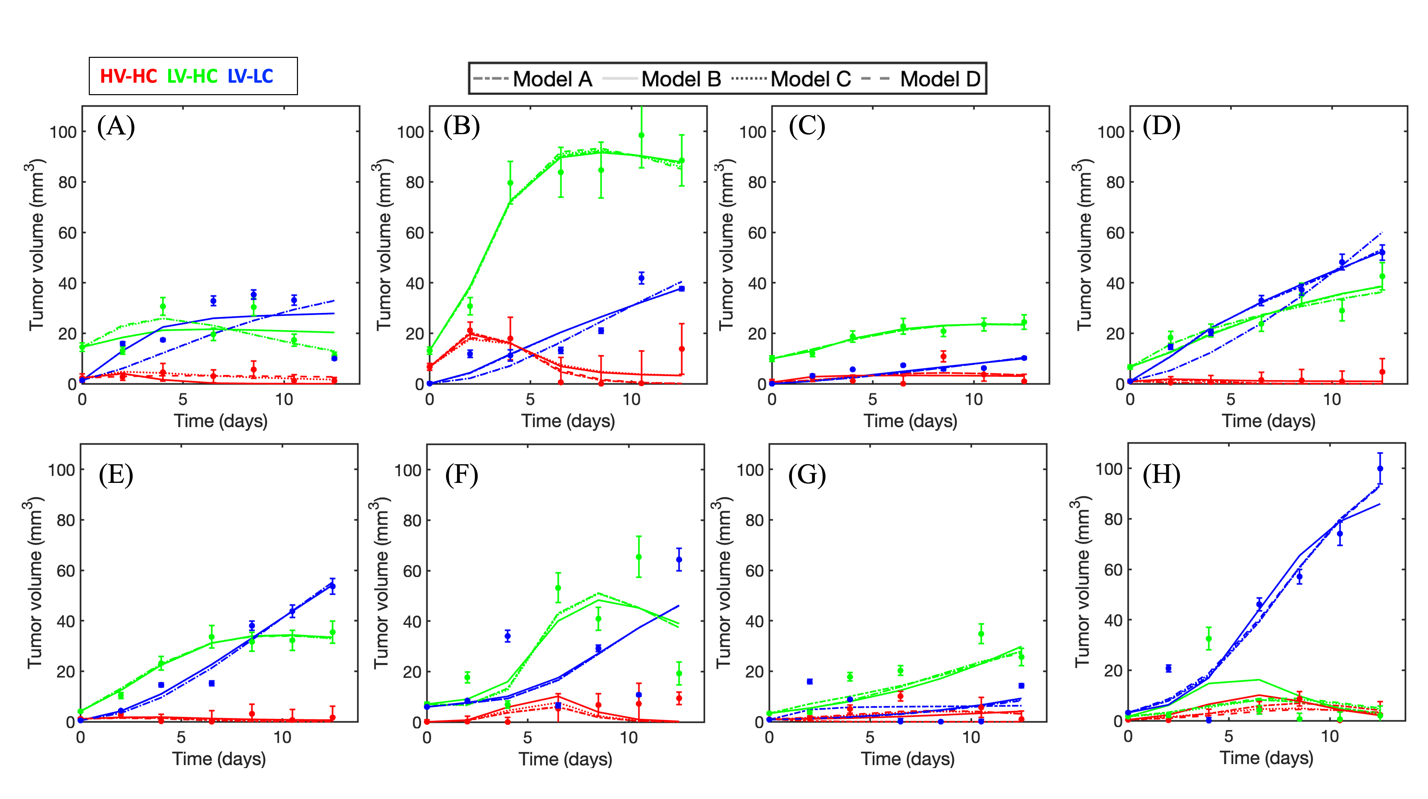


**Figure S6: A family of four models calibrated to the habitat time series of eight treated rats.** Each panel displays the model calibrations for a particular rat dataset from the treated cohort of rats that were selected for the modelling analysis (See Table S1 for each rat’s imaging schedule and radiation dose, where (A) is rat R1, (B) is rat R2, and so on). Data are represented by solid circles with confidence intervals computed as described in the Methods. Red, green, and blue correspond to the HV-HC, LV-HC, and LV-LC habitats, respectively. Each model is represented by a different line style as depicted in the legend at the top of the figure. There is high similarity between the fits of all four models across all rats, suggesting that each model shows similar performance in describing the dynamics of each habitat time series.


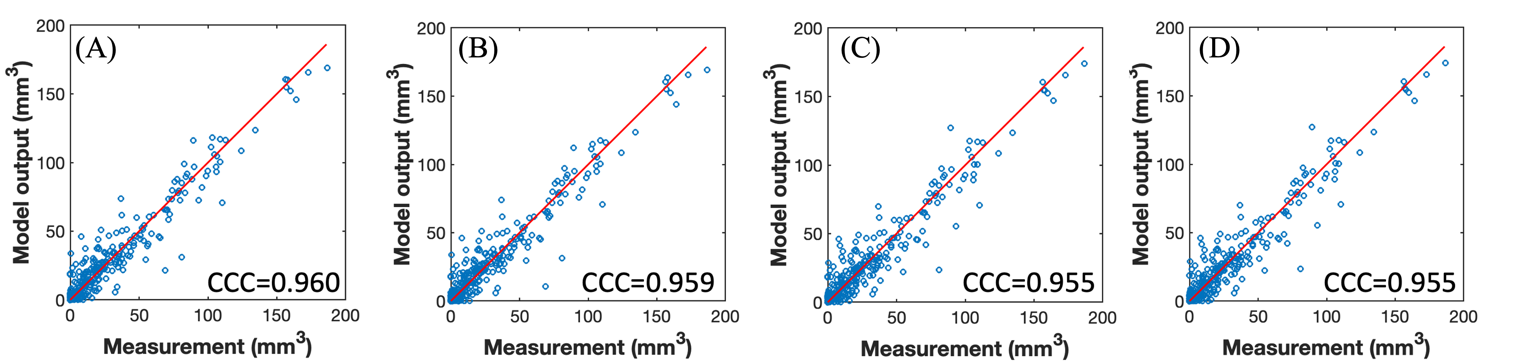


**Figure S7: Evaluating the agreement between the model outputs and the measured habitat volumes**. (A) Plot of the parent model (Model A) outputs on the *y*-axis and the habitat measurements on the *x*-axis, where the model outputs and the corresponding data points are pooled from all three habitats and plotted collectively. The concordance correlation coefficient (CCC) is visible in the lower right corner of the panel, where CCC>0.8 indicates strong agreement. (B)-(C) similar to panel (A) but for Models B, C, and D, respectively. All four models strongly agree with the measured data (CCC>0.950).


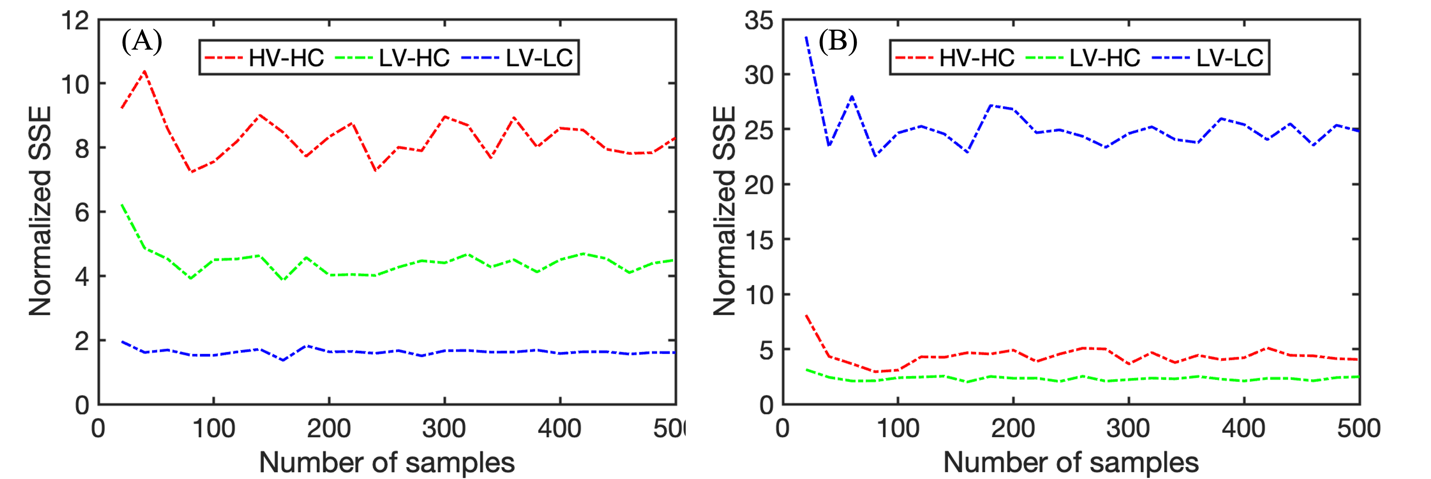


**Figure S8: Plotting the SSE as a function of the number of samples, *N_b_*, in bootstrapping.** (A) SSE as a function of *N_b_* for the control rat cohort, where red represents the HV-HC habitat data points, green represents the LV-HC datapoints, and blue represents the LV-LC datapoints in blue. (B) Analogous to the information in panel (A) but for the treated rat cohort. An appropriate value for *N_b_* was selected by qualitatively inspecting these plots for a stable trend in the SSE values.

Supplemental Tables

| **Rat** | **Imaging visits as days post-inoculation** | **Radiation dose (Gy)** |
| --- | --- | --- |
| **C1** | 10 12 14 15 16 18 20 | 0 |
| **C2** | 10 12 14 15 16 18 20 | 0 |
| **C3** | 10 12 14 15 16 18 | 0 |
| **C4** | 10 12 14 15 16 18 | 0 |
| **C5** | 10 12 14 15 16 18 20 | 0 |
| **C6** | 10 12 14 15 16 | 0 |
| **C7** | 10 12 14 15 16 18 20 | 0 |
| **C8** | 10 12 14 15 16 18 | 0 |
| **R1** | 10 12 14 [RT] 16 18 20 22 | 40 |
| **R2** | 10 12 14 [RT] 16 18 20 22 | 40 |
| **R3** | 10 12 14 [RT] 16 18 20 22 | 40 |
| **R4** | 10 12 14 [RT] 16 18 20 22 | 20 |
| **R5** | 10 12 14 [RT] 16 18 20 22 | 20 |
| **R6** | 10 12 14 [RT] 16 18 20 22 | 20 |
| **R7** | 10 12 14 [RT] 16 18 20 22 | 40 |
| **R8** | 10 12 14 [RT] 16 18 20 22 | 20 |

**Table S1**. Imaging schedule for each rat. Rats in the control group (C) did not receive radiation therapy, shown as 0 Gray (Gy), whereas rats in the treated cohort (R) received the specified amount of radiation therapy in a single dose delivered between the third and fourth imaging visits, denoted as “[RT]”.

| Habitat | *k_ep_* | *v_e_* | *K^trans^* | *ADC* |
| --- | --- | --- | --- | --- |
| HV-HC | 1.625 (±0.788) min^-1^ | 0.232 (±0.158) | 0.307 (±0.155) min^-1^ | 0.826×10^-3^ (±0.216) mm^2^/s |
| LV-HC | 0.447 (±0.310) min^-1^ | 0.181 (±0.118) | 0.070 (±0.054) | 0.852×10^-3^ (±0.186) mm^2^/s |
| LV-LC | 0.029 (±0.097) min^-1^ | 0.848 (±0.199) | 0.0276 (±0.034) min^-1^ | 0.945×10^-3^ (±0.280) mm^2^/s |

**Table S2**. Mean (± standard deviation) of the MRI parameter values for each habitat. All MRI parameter distributions were found to be significantly different between the three habitats with *p* < 0.0001.

| _Rat_^Param^ | ***k_1_*** | ***d_12_*** | ***d_21_^*^*** | ***d_13_^*^*** | ***k_2_*** | ***d_23_*** | ***𝛉 ^*^*** |
| --- | --- | --- | --- | --- | --- | --- | --- |
| **C1** | 1.560  (-4.83, -5.99) | 0.594 (-5.99,7.18) | 0.082  (-0.33, 0.50) | 0.407  (-0.22, 1.03) | 0.158  (-4.18, 4.50) | **0**  (-0.20, 0.20) | 371.952  (-673.13, 1417) |
| **C2** | 1.835  (-5.48, 9.15) | 1.405  (-4.70, 7.51) | **0**  (-0.62, 0.62) | **0**  (-1.08, 1.08) | 0.001  (-3.02, 3.02) | 0.253  (-0.08, 0.59) | 907.337  (-5.34, 7.16)×10^-3^ |
| **C3** | 1.034  (-20.99, 23.05) | 0.582  (-21.30, 22.46) | **0**  (-0.35, 0.35) | **0**  (-0.58, 0.58) | 0.163  (-16,81, 17.14) | 0.104  (-0.16, 0.37) | 448.258  (-9.41, 1.03)×10^-3^ |
| **C4** | 0  (-7.90, 7.90) | 0.037  (-12.54, 12.62) | 0.008  (-0.42, 0.44) | 0.071  (-4.74, 4.88) | 0.368  (-0.30,1.04) | 0.193  (-0.12, 0.51) | 581.421  (-345, 1.51×10^-3^) |
| **C5** | 2.766  (-0.97, 6.50) | 1.892  (-1.55, 5.34) | **0**  (-0.17, 0.17) | **0**  (-0.74, 0.74) | **0**  (-1.41, 1.41) | 0.182  (-0.01, -0.38) | 574.501  (-547, 1.70×10^-3^) |
| **C6** | 2.801  (1.45, 4.16) | 0.247  (-0.37, 0.86) | **0**  (-0.65, 0.65) | 0.052  (-0.29, 0.40) | **0**  (-3.28, 3.28) | **0**  (-0.51, 0.51) | 46.25  (34, 58) |
| **C7** | 1.073  (-9.61, 11.75) | 0.608  (-9.50, 10.72) | **0**  (-0.76, 0.76) | **0**  (-1.31, 1.31) | **0**  (-10.28, 10.28) | 0.175  (-0.60, 0.95) | 282.762  (-2.87, 3.43)×10^-3^ |
| **C8** | 1.852  (-7.39, 11.10) | 0.867  (-8.25, 9.99) | **0**  (-0.58, 0.58) | 0.308  (-1.80, 2.41) | 0.053  (-4.29, 4.40) | **0**  (-0.71, 0.71) | 506.545  (-2.47, 3.48)×10^-3^ |
| **R1** | 3.823  (-1.45, 9.10) | 0.590  (-3.37, 4.55) | **0**  (-0.32, 0.32) | 1.570  (-4.40, 7.54) | **0**  (-1.61, 1.61) | 0.012  (-0.60, 0.62) | 73.944  (-239, 387) |
| **R2** | 2.148  (-0.53, 4.82) | 0.956  (-2.37, 4.28) | 0.031  (-0.07, 0.13) | 0.111  (-0.11, 0.33) | 0.072  (-2.73, 2.87) | 0.027  (-0.01, 0.06) | 141.488  (-144, 427) |
| **R3** | 2.516  (-27.70, 32.73) | 4.242  (-61.44, 69.92) | 0.624  (-8.60, 9.85) | 0.261  (-3.55, 4.07) | 0.012  (-6.04, 6.06) | **0**  (-0.61, 0.61) | 38.963  (12, 65) |
| **R4** | 5.000  (-3.07, 13.07) | 1.237  (-4.54, 7.02) | 0.070  (-0.31, 0.45) | 3.265  (-2.15, 8.68) | 0.211  (-0.71, 1.14) | **0**  (-0.41, 0.41) | 136.118  (-94, 366) |
| **R5** | 3.751  (-7.53, 15.03) | 2.953  (-9.41, 15.32) | 0.024  (-0.33, 0.38) | 0.487  (-0.54, 1.52) | 0.167  (-1.98, 2.32) | 0.143  (-0.06, 0.22) | 191.918  (-1.99, 2.37) ×10^-3^ |
| **R6** | 3.961  (-7.31, 15.23) | 2.499  (-12.19, 17.18) | **0**  (-0.19, 0.19) | **0**  (-2.37, 2.37) | **0**  (-1.92, 1.92) | 0.106  (-0.12, 0.33) | 143.298  (-709, 996) |
| **R7** | 1.420  (-78.70, 81.54) | 1.747  (-96.12, 99.62) | 0.167  (-6.24, 6.57) | 0.006  (-6.35, 6.36) | 0.038  (-21.11, 21.18) | 0.045  (-1.19, 1.28) | 74.018  (-2.60, 2.75)×10^-3^ |
| **R8** | 0.004  (-205.30,205.30) | 0.062  (-26.80, 26.93) | 0.668  (-87.88,89.22) | 1.274  (-30.62, 33.17) | 1.669  (-74.11, 77.45) | 0.619  (-15.11, 16.35) | 250.268  (-1.03, 1.08) ×10^-3^ |

**Table S3. Calibrated model parameters** **for the parent model**. The confidence intervals are displayed in parentheses under each parameter value. The asterisks next to the parameters *d_21_*, *d_13_*, and *θ* indicate significant differences (*p*<0.05) in the values of these parameters between the control (C) and treated (R) rats.

| **Rat** | **Model A** | **Model B** | **Model C** | **Model D** |
| --- | --- | --- | --- | --- |
| **C1** | -29.616 | -34.285 | -34.282 | **-52.27** |
| **C2** | -34.308 | -39.005 | -39.007 | **-57.005** |
| **C3** | -30.164 | -34.161 | -34.832 | **-52.162** |
| **C4** | -29.758 | -34.425 | -34.425 | **-52.425** |
| **C5** | -30.994 | -35.673 | -35.444 | **-53.453** |
| **C6** | -32.334 | -37.000 | -36.612 | **-54.612** |
| **C7** | -27.593 | -32.582 | -32.539 | **-50.539** |
| **C8** | -24.526 | -29.195 | -29.186 | **-47.186** |
| **R1** | -36.631 | -32.055 | -36.256 | **-54.255** |
| **R2** | -36.548 | -31.881 | -36.182 | **-54.182** |
| **R3** | -39.433 | -34.766 | -39.433 | **-57.433** |
| **R4** | -39.335 | -34.71 | -37.011 | **-55.013** |
| **R5** | -38.428 | -33.871 | -38.443 | **-56.427** |
| **R6** | -32.579 | -27.913 | -32.578 | **-50.579** |
| **R7** | -34.066 | -29.395 | -34.123 | **-52.08** |
| **R8** | -32.680 | -28.218 | -32.657 | **-50.68** |

**Table S4.** AIC values for all four models calibrated to the data from all 16 rat datasets. Model D consistently had the lowest AIC value and was selected as the most parsimonious model.

| _Rat_^Param^ | ***k_1_*** | ***d_12_*** | ***k_2_^*^*** | ***d_23_*** | ***𝛉 ^*^*** |
| --- | --- | --- | --- | --- | --- |
| **C1** | 0.303  (00.68, 1.29) | 0  (-0.60, 0.60) | 0.557  (0.32, 0.79) | 0.125  (0.08, 0.17) | 420.469  (220, 621) |
| **C2** | 1.834  (-1.84, 5.51) | 1.403  (-2.44, 5.25) | 0  (-1.45, 1.45) | 0.253  (0.21, 0.30) | 905.012  (1.76, 3.57)×10^-3^ |
| **C3** | 1.033  (-12.74, 14.80) | 0.584  (-13.20, 14.37) | 0.162  (-10.30, 10.62) | 0.105  (0.07, 0.14) | 451.240  (-5.67, 6.57)×10^-3^ |
| **C4** | 3.168  (-11.30, 17.63) | 2.897  (-12.17, 17.98) | 0  (-1.55, 1.56) | 0.200  (0.18, 0.22) | 1966.695  (01.38, 1.78)×10^-3^ |
| **C5** | 2.766  (0.11, 5.43) | 1.893  (-0.96, 4.74) | 0  (-1.00, 1.00) | 0.182  (0.15, 0.21) | 574.600  (0257, 1.40×10^-3^) |
| **C6** | 2.788  (2.19, 3.39) | 0.286  (0.07, 0.51) | 0  (-2.78, 2.78) | 0.056  (-.04, 0.16) | 44.893  (35, 55) |
| **C7** | 1.073  (-6.61, 8.76) | 0.608  (-7.33, 8.55) | 0  (-7.38, 7.38) | 0.175  (0.08, 0.27) | 283.163  (-2.02, 2.59)×10^-3^ |
| **C8** | 1.829  (-16.26, 19.92) | 1.276  (-17.08, 19.64) | 0.079  (-6.20, 6.36) | 0.093  (-0.01, 0.19) | 786.897  (-7.97, 9.64) ×10^-3^ |
| **R1** | 0.263  (-4.76, 5.29) | 0.016  (-0.91, 0.94) | 0.750  (-0.26, 1.76) | 0.122  (0.06, 0.18) | 48.764  (28, 69) |
| **R2** | 1.872  (-1.48, 5.22) | 0.861  (-2.99, 4.71) | 0.150  (-3.10, 3.35) | 0.044  (0.03, 0.06) | 137.022  (-172, 446) |
| **R3** | 0.899  (-0.14, 1.93) | 0.139  (-0.16, 0.44) | 0.288  (0.13, 0.44) | 0.043  (0.03, 0.06) | 39.414  (28, 51) |
| **R4** | 4.892  (-4.75, 14.53) | 5.000  (-5.76, 15.76) | 0.280  (-0.80, 1.37) | 0.186  (0.14, 0.23) | 395.557  (-5.91, 6.70)×10^-3^ |
| **R5** | 3.381  (-4.67, 11.43) | 3.104  (-6.27, 12.47) | 0.240  (-0.92, 1.40) | 0.170  (-0.15, 0.19) | 235.747  (-1.75, 2.22)×10^-3^ |
| **R6** | 3.972  (-6.05, 14.00) | 2.508  (-9.17, 14.18) | 0  (-0.75, 0.75) | 0.106  (-0.04, 0.17) | 143.425  (-368, 655) |
| **R7** | 0.751  (-3.80, 5.30) | 0.376  (-3.33, 4.08) | 0.184  (-1.71, 2.08) | 0.043  (-0.03, 0.12) | 55.630  (-105, 216) |
| **R8** | 1.080  (-1.70, 2.86) | 0.572  (-1.45, 2.59) | 1.415  (0.38, 2.45) | 1.135  (-0.19, 2.46) | 165.943  (-60, 392) |

**Table S5. Calibrated model parameters** **for the most parsimonious model, Model D**. The confidence intervals are displayed in parentheses under each parameter value. The asterisks next to the parameters *k_2_* and *θ* indicate significant differences (*p*<0.05) in the values of these parameters between the control (C) and treated (R) rats.
